# Prediction of Novel Trigonal Chloride Superionic Conductors as Promising Solid Electrolytes for All‐Solid‐State Lithium Batteries

**DOI:** 10.1002/advs.202404213

**Published:** 2024-07-09

**Authors:** Yao Wang, Ziang Ren, Jinsen Zhang, Shaohua Lu, Chenqiang Hua, Huadong Yuan, Jianmin Luo, Yujing Liu, Jianwei Nai, Xinyong Tao

**Affiliations:** ^1^ College of Materials Science and Engineering Zhejiang University of Technology Hangzhou 310014 China; ^2^ Moganshan Research Institute at Deqing County Zhejiang University of Technology Huzhou 313000 China; ^3^ Collaborative Center for Physics and Chemistry Institute of International Innovation Beihang University Hangzhou 311115 China

**Keywords:** all‐solid‐state batteries, cation ordering, chloride solid‐state electrolytes, first‐principles calculations, structure prediction method

## Abstract

Recently emerging lithium ternary chlorides have attracted increasing attention for solid‐state electrolytes (SSEs) due to their favorable combination between ionic conductivity and electrochemical stability. However, a noticeable discrepancy in Li‐ion conductivity persists between chloride SSEs and organic liquid electrolytes, underscoring the need for designing novel chloride SSEs with enhanced Li‐ion conductivity. Herein, an intriguing trigonal structure (i.e., Li_3_SmCl_6_ with space group *P*3_1_12) is identified using the global structure searching method in conjunction with first‐principles calculations, and its potential for SSEs is systematically evaluated. Importantly, the structure of Li_3_SmCl_6_ exhibits a high ionic conductivity of 15.46 mS cm^−1^ at room temperature due to the 3D lithium percolation framework distinct from previous proposals, associated with the unique in‐plane cation ordering and stacking sequences. Furthermore, it is unveiled that Li_3_SmCl_6_ possesses a wide electrochemical window of 0.73−4.30 V vs Li^+^/Li and excellent chemical interface stability with high‐voltage cathodes. Several other Li_3_MCl_6_ (M = Er, and In) materials with isomorphic structures to Li_3_SmCl_6_ are also found to be potential chloride SSEs, suggesting the broader applicability of this structure. This work reveals a new class of ternary chloride SSEs and sheds light on strategy for structure searching in the design of high‐performance SSEs.

## Graphical abstract

In this research, using the global structure searching method in conjunction with first‐principles calculations, a new chloride superionic conductor, Li_3_SmCl_6_, is identified, characterized by its unique structure and high ionic conductivity. With its robust ionic performance and broad electrochemical stability, Li_3_SmCl_6_ emerges as a promising candidate for next‐generation solid‐state electrolytes.

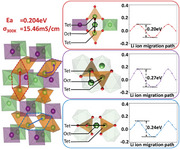

## 1 Introduction

Current novel energy storage technologies predominantly hinge on conventional lithium‐ion batteries, which have significant advantages in terms of energy density, energy utilization, and technological maturity.^[^ ^1^ ^]^ However, lithium‐ion batteries are approaching their energy density limits, and there are still unresolved safety issues, particularly regarding the risk of combustion.^[^ ^2^ ^]^ Solid‐state electrolytes (SSEs) are emerging as a promising solution to address the issues of safety and energy density in lithium‐ion batteries. Many global entities, from governments to automotive giants and battery manufacturers, are now focusing on the development of all‐solid‐state lithium metal batteries due to their high energy density and mechanical robustness that inhibits the growth of lithium dendrites.^[^ ^3^, ^4^, ^5^, ^6^ ^]^

Among the myriad SSEs being researched, sulfide SSEs stand out due to their exceptional ionic conductivity. Notably, Li_10_GeP_2_S_12_ and Li_7_P_3_S_11_ exhibit impressive ionic conductivities reaching up to 12 and 17 mS cm^−1^, respectively,^[^ ^7^, ^8^ ^]^ which are comparable to that of conventional organic liquid electrolytes. Nevertheless, sulfide SSEs suffer from inadequate chemical stability with a narrow electrochemical window between 1.7 and 2.1 V, and pose challenges in terms of processability. On the one hand, oxide SSEs generally exhibit commendable ionic conductivity (1.7 mS cm^−1^ for Li_7_La_3_Zr_2_O_12_^[^ ^9^ ^]^) but grapple with issues related to rigid interfacial contact, severe side reactions, and fabrication complexities.^[^ ^10^, ^11^, ^12^ ^]^ On the other hand, polymer SSEs are praised for their exemplary interfacial compatibility and mechanical processability.^[^ ^13^ ^]^ However, their ionic conductivity at room temperature (RT) remains lackluster (10^‐5^–10^‐3^ mS cm^−1^ for polyethylene oxide^[^ ^14^ ^]^), which limits their operational temperature range. In recent academic discourses, lithium ternary chlorides have gained traction for SSEs applications, owing to their favorable combination between lithium ionic conductivity, electrochemical stability, and chemical compatibility with cathodes.^[^ ^15^ ^]^ Notably, Li_0.388_Ta_0.238_La_0.475_Cl_3_ can achieve a high lithium ionic conductivity up to 3.02 mS cm^−1^ at RT.^[^ ^16^ ^]^ A fundamental understanding of the lithium‐ion diffusion mechanism is crucial for guiding in the exploration and designing novel chloride SSEs. Given that most chloride electrolytes are composed of ionic materials, which can be classified into cation sublattice and anion sublattice, the previous study focused primarily on the modulation strategy for both sublattices. For the anion sublattice, the cubic‐close‐packing (ccp) structure enables isotropic ionic diffusion, contrasting with the hexagonal close‐packed (hcp) structure, which leads to anisotropic diffusion and may also contribute to blocking effects.^[^ ^15^, ^17^ ^]^ Conversely, the cation sublattice benefits from low cation concentration and sparse distribution or from cation disordering, each influencing the ionic conduction paths distinctly.^[^ ^18^, ^19^, ^20^ ^]^ Beyond these results, a zeolite‐like SmCl_3_ frameworks achieve an ionic conductivity over 0.12 mS cm^−1^ at RT.^[^ ^21^ ^]^ Considering the aforementioned findings, it is reasonable to speculate that there exist undiscovered chloride superionic conductor structures with significant potential.

In this study, using crystal structure prediction in combination with first‐principles calculations, we identify a novel chloride superionic conductor, Li_3_SmCl_6_ (LSC), with ccp anion sublattice and distinctive cation ordering of stacking sequences in trigonal structure. The LSC features a *P*3_1_12 space group symmetry with cations adopting an ABC stacking pattern, where each layer rotationally shifts by 120° to maintain the sequence continuity. The LSC structure is noteworthy for its high ionic conductivity of 15.46 mS cm^−1^ at RT, which can be compared with that of the organic liquid electrolytes. This result can be attributed to its unique in‐plane cation ordering and stacking sequences. In addition, we reveal that LSC has a broad electrochemical range of 0.73−4.30 V compared to Li^+^/Li and demonstrates exceptional chemical interface stability when used with high‐voltage cathodes. The Sm component of LSC structure can be substituted with metal element Er and In, which likewise exhibit the features of chloride SSEs with promising performance. This work uncovers a novel category of chloride structures that function as SSEs. A thorough exploration to comprehend the superior performance of these new chloride systems will provide rational guidance for the design of innovative SSEs and valuable insights for laboratory synthesis.

## 2 Results

**Figure 1:**
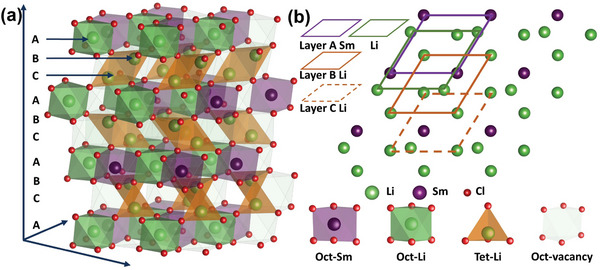
a) Proposed structure of LSC with *P*3_1_12 space group, created by the ABCABC stacking order of the metal cation layers. b) [001]_hex._ projection of cation layers reveals the structured stacking sequence along the *c* axis.

The entire workflow of this study is illustrated in Figure S1 (Supporting Information). The crystal LSC adopts a trigonal *P*3_1_12 space group, where Sm atoms occupy the 3a Wyckoff positions and exhibit a layered‐type structure, as shown in **Figure** **1**. There is an alternating stratification of two kinds of distinct layers: Sm layer (Figure 1a, A layer) encompassing both SmCl_6_ and LiCl_6_ octahedra, punctuated by vacant octahedral sites, and Li layer (Figure 1a, B and C layer) which contains LiCl_4_ tetrahedra and some vacant octahedral and tetrahedral sites, two asymmetrically distinct Li^+^ sites are identified: 3a and 6c. The Li^+^ in the 3a site are 6‐coordinated and localized within the Sm layer, while the Li^+^ in the 6c site is 4‐coordinated, and localized within the Li layer. The cation in‐plane ordering remains consistent with the *C*2/*m* space group in Li_3_YBr_6_, but exhibits distinct stacking sequences.^[^ ^15^ ^]^ Figure 1b illustrates the [001] hexagonal projection of these layers, clearly delineating the positioning of Sm and Li within the lattice. The space group*P*3_1_12 holds a threefold screw axis along the *c* axis while the *C*2/*m* has a twofold axis. The ccp anion sublattice is identical to that of Li_3_YBr_6_ and may lead to isotropic lithium diffusion. And given that the trigonal structures of lithium chloride solid electrolytes, such as Li_3_YCl_6_ and Li_3_ErCl_6_ with space group ***P*** $\bar{3}m1$, have been previously reported.^[^ ^15^ ^]^ In this study, we conducted a comparison between LSC with space group *P*3_1_12 (this work) and LSC derived from element substitution of Li_3_YBr_6_ and Li_3_YCl_6_ with space group *C*2/*m* and ***P*** $\bar{3}m1$, as shown in Figure S2 (Supporting Information). From the perspective of cation coordination, both *C*2/*m* and ***P*** $\bar{3}m1$ LSCs are composed of 6‐coordinated LiCl_6_ and SmCl_6_ octahedra, whereas the *P*3_1_12 LSC not only includes these octahedra on the same plane but also features LiCl_4_ tetrahedra. Regarding the stacking sequence, the *C*2/*m* and ***P*** $\bar{3}m1$ LSC exhibit an ABAB stacking pattern, while the *P*3_1_12 LSC adopts an ABCABC stacking pattern. Therefore, although this crystal shares the trigonal phase with previously reported Li_3_ErCl_6_ and the ccp anion sublattice arrangement with Li_3_YBr_6_, their crystal structures are entirely different. We have also included the 3D electron localization function maps to showcase the electronic structure of LMC, as illustrated in Figures S3 and S4 (Supporting Information). It is worth noting that the coexistence of tetrahedral Li (T‐Li) and octahedral Li (O‐Li) in layered structures has been rarely observed previously but is thermodynamically plausible because the type of coordinating anions and lattice volume couple effect would affect the stable Li site geometry.^[^ ^22^ ^]^ These MCl_6_ (M = Li, Sm) octahedra interlink in an edge‐sharing manner, as do the LiCl_4_ tetrahedra, but MCl_6_ (M = Li, Sm) octahedra and LiCl_4_ tetrahedra from different layers are connected in a corner‐sharing manner. The distinctive interconnection results in a continuous 3D lattice that facilitates diffusion, the implications of which on Li^+^ diffusion will be explored subsequently.

**Figure 2:**
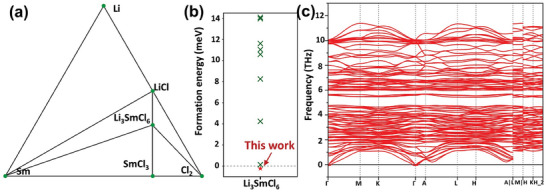
a) Phase diagram of LSC. b) Calculated formation energy of different structure of LSC. c) The phonon spectrums of LSC.

Prior to assessing the viability of LSC as SSE, it is crucial to thoroughly examine its stability from many perspectives. The thermodynamic stability of LSC was evaluated using a phase diagram from the Materials Project database, with the phase stability quantified by the calculated energy above hull (Δ*E*_above_hull_) against competing stable phases,^[^ ^23^ ^]^ as depicted in **Figure** **2**a. Our results reveal that LSC possesses a negative Δ*E*_above_hull_ value (where a negative value indicates a lower energy relative to the previously established convex hull) (≈0.6 meV per atom), indicating its inherent structural stability. The calculated formation energy of LSC, relative to the other configurations depicted in Figure 2b, also demonstrate its energy stability. We also calculated temperature‐dependent free energy for LSC across various space groups: *P*3_1_12 (trigonal, this work), *C*2/*m* (monoclinic), ***P*** $\bar{3}m1$ (trigonal), and *Pnma* (orthorhombic). These calculations aim to elucidate the temperature‐dependent structural stability from 0 to 1000 K, as shown in Figure S5 (Supporting Information). The results reveal that the *P*3_1_12 phase of LSC exhibits the highest stability in the 0–375 K temperature range. This implies that at room temperature, the LSC with space group *P*3_1_12 is thermodynamically favored. Additionally, we performed AIMD simulations at 600 K to investigate the thermal stability of LSC. Figure S6 (Supporting Information) illustrates the total energy evolution during the simulation time (20 ps), the value of potential energy remains constant with negligible fluctuation. The snapshot of LSC at 20 ps exhibits no structural perturbations in thermal equilibrium, thereby confirming its thermal stability. Furthermore, the dynamic stability of LSC was verified through phonon calculations, and the phonon dispersion spectra are shown in Figure 2c. Only a little imaginary frequency (0.06 THz) is presented in the vicinity of the Γ point over the whole Brillouin zone, suggesting the dynamic stability.^[^ ^24^ ^]^ Subsequently, to study the universality of the LMC framework, we adopted the same structure and constructed numerous chloride materials LMC with different metal ions framework (M = Sc, Y, In, La, Ce, Sm, Gd, Tb, Dy, Er, Tm and Yb), and their respective Δ*E*_above_hull_ values were systematically evaluated. The LMC configurations with positive Δ*E*_above_hull_ values of up to 25 meV per atom were defined as thermodynamically metastable phases, which might potentially be synthesized at RT stabilized by entropic effects.^[^ ^25^, ^26^ ^]^ As shown in Figure S7 (Supporting Information), although Sc, La, Ce, and Yb deviate from the metastable phase criterion of determination, most of the metal ions display a low Δ*E*_above_hull_ of approximately 25 meV per atom. Notably, the elements In and Er even exhibit negative Δ*E*_above_hull_ values. These findings demonstrate the expansive potential and adaptability of the LMC framework, highly desirable for optimizing the performance of chloride Li‐superionic conductors. We chose In and Er substituted compounds for further investigation of their phonon spectra and thermal stability, and the results suggest that Li_3_ErCl_6_ (LEC) and Li_3_InCl_6_ (LIC) possess stable structures and could be candidates for chloride SSEs, as shown in Figures S6, S8, and S9 (Supporting Information). The crystal structures of LEC and LIC display a high degree of similarity to LSC, with only minimal variations in their lattice constants.

**Figure 3:**
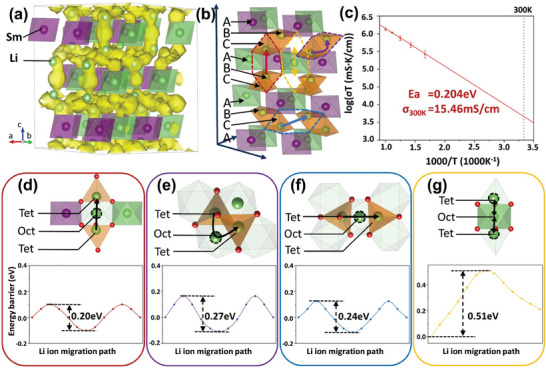
a) Crystal structures of LSC, superimposed with Li^+^ probability density marked by yellow isosurfaces from AIMD simulations. b) The Li^+^ migration pathways in LSC, with pathways indicated by dashed lines in four different colors. c) Arrhenius plot of Li^+^ diffusivity in LSC from AIMD simulations. d–g) Four particular Li^+^ migration pathways with migration barriers, each corresponding to one of the colored dashed lines in panel (b).

To better evaluate the performance of this novel chloride SSE, we carried out AIMD simulations to investigate Li^+^ diffusion behavior within LSC, LEC, and LIC materials. **Figure** **3**a illustrates the Li^+^ probability density superimposed on the anion sublattice, and the schematic of the 3D diffusion pathways of Li^+^ is depicted in Figure 3b. The calculation results verify the facile Li^+^ diffusion in the continuous 3D channels present in these three materials. AIMD simulations were conducted at temperatures ranging from 600 to 1000 K with an interval of 100 K, and the Arrhenius diagram of Li^+^ diffusivity in LSC at various temperatures is illustrated in Figure 3c. The activation energy (*E*_a_) of LSC is 0.20 ± 0.01 eV and its extrapolated conductivity at 300 K (*σ*_RT_) is 15.46 mS cm^−1^. For LEC and LIC, the AIMD simulations estimated *σ*_RT_ to be 7.23 mS cm^−1^ with lower bounds of 3.98 and 5.25 mS cm^−1^ with a lower bound of 0.36 mS cm^−1^, respectively (Figure S10, Supporting Information). As listed in **Table** **1**, the ionic conductivity value of 15.46 mS cm^−1^ for LSC is higher than the values reported for previously superionic conductors such as Li_3_YCl_6_ (14 mS cm^−1^), Li_3_YBr_6_ (2.2 mS cm^−1^), Li_3_ScBr_6_ (1.4 mS cm^−1^), Li_3_HoBr_6_ (3.8 mS cm^−1^), Li_10_GeP_2_S_12_ (12 mS cm^−1^) and Li_7_La_3_Zr_2_O_12_ (1.7 mS cm^−1^),^[^ ^7^, ^9^, ^15^ ^]^ which indicates that the novel LSC structure has excellent Li^+^ diffusion features. MSD plots for AIMD simulations are shown in Figures S11–S13 (Supporting Information).

**Table 1:** Calculated Li^+^ conductivities and activation energies for different materials from AIMD simulations in comparisons of the experimental (Expt.) values at 300 K.

Composition	*σ* at 300 K [mS cm^−1^]		Error bound [σ_min_, *σ*_max_] [mS cm^−1^]	*E*_a_	
	AIMD	Expt.		AIMD	Expt.
Li_3_SmCl_6_ (this work)	15.46	/	[8.87, 26.92]	0.20 ± 0.01	/
Li_3_ErCl_6_ (this work)	7.23	/	[3.98, 13.13]	0.24 ± 0.05	/
Li_3_InCl_6_ (this work)	5.25	/	[0.36, 75.49]	0.25 ± 0.01	/
Li_3_ErCl_6_	3^[^ ^27^ ^]^	0.33^[^ ^28^ ^]^	/	<0.4^[^ ^27^ ^]^	0.41^[^ ^28^ ^]^
Li_3_InCl_6_	21^[^ ^5^ ^]^	2.04^[^ ^29^ ^]^	/	0.2^[^ ^5^ ^]^	0.35^[^ ^29^ ^]^
Li_3_YCl_6_	14^[^ ^15^ ^]^	0.5	[4.5, 47]	0.19 ± 0.03	0.40
Li_10_GeP_2_S_12_	12^[^ ^30^ ^]^	12^[^ ^7^ ^]^	[8.1, 25]	0.17^[^ ^30^ ^]^	0.25^[^ ^7^ ^]^
Li_7_La_3_Zr_2_O_12_	2.1^[^ ^31^ ^]^	1.7^[^ ^9^ ^]^	[0.5, 2.1]	0.24^[^ ^31^ ^]^	0.27^[^ ^9^ ^]^

The unique framework structure of LSC (i.e., ccp anion sublattice and distinctive cation ordering of stacking sequences) gives rise to a different Li^+^ diffusion mechanism and pathway compared to other chloride SSEs, as illustrated in Figure 3a,b. The trajectory of Li^+^ diffusion observed in AIMD simulations aligns with the pathways determined by the BVEM method. Based on the AIMD simulations and supplemented by BVEM analysis, we further explored the distinct Li^+^ migration pathways in LSC. We identified four distinct Li^+^ migration pathways within the material, each presenting different Li^+^ diffusion properties and migration barriers: i) Along the *c*‐axis, Li^+^ migrates between separate lithium layers. Specifically, tetrahedral Li^+^ (T‐Li) in the B layer (depicted as orange tetrahedra) moves through an adjacent empty octahedral site in the A layer (illustrated as light green octahedra) to another T‐Li in the C layer, overcoming a diffusion barrier of 0.20 eV (Figure 3d). Within the *ab*‐plane, specifically in the same Li layer (B layer and adjacent C layer), Li^+^ migration can occur between tetrahedral sites (T‐Li) in layers B and C. Specifically, Li^+^ moves from a T‐Li site in layer B to a corresponding T‐Li site in layer C, passing through an intervening vacant octahedral site. This process can also occur in the reverse direction. It is essential to distinguish between two migration pathways: ii) migration through edge‐sharing tetrahedral Li (T‐Li) sites across adjacent B and C layers, with an associated energy barrier of 0.27 eV (Figure 3e), and iii) migration between non‐adjacent T‐Li sites spanning B and C layers, which encounters a slightly lower energy barrier of 0.24 eV (Figure 3f). iv) In the context of the O‐Li ions located at the green octahedral sites within the A layer, we have investigated its transport along the *c*‐axis direction. Specifically, the O‐Li transitions to an adjacent empty tetrahedral site within the Li layers (Figure 3g). However, the presence of a considerable energy barrier, quantified at 0.51 eV, between the O‐Li and the targeted vacant tetrahedral site suggests that this migration pathway is significantly less frequent compared to other migrations occurring between different Li layers. The Li^+^ occupying 3a Wyckoff positions in Sm layer donates little to Li^+^ migration, which means that 3a‐Li (denoted as O‐Li) is mainly used for forming the rhombus skeleton while 6c‐Li (T‐Li) contributes to the Li^+^ diffusivity. The *P*3_1_12 LSC's unique triple helix axis eliminates straight 1D channels along the *c*‐axes. After a brief transition in the c direction, Li^+^ transport must occur via LiCl_4_ tetrahedra within the ab plane. We can find that the primary migration pathway for Li^+^ is “T‐Li→O→T‐Li” route, a mechanism distinct from both the *C*2/*m* and ***P*** $\bar{3}m1$ LSCs, where octahedral Li moves through tetrahedral Li to a new octahedral Li (O‐Li→T→O‐Li), or directly from octahedral Li to another octahedral Li (O‐Li→O‐Li). Therefore, although this crystal shares the trigonal phase with previously reported LEC and the ccp anion sublattice arrangement with Li_3_YBr_6_, its crystal structure and ionic transport mechanism are entirely different. Additionally, the strong time correlation in Li^+^ hopping during the concerted migration is confirmed by the van Hove correlation function, as shown in Figure S14 (Supporting Information). The results of LEC and LIC's AIMD simulations and BVEM analyses are similar to LSC's, indicating that they share identical Li^+^ migration pathways. In pursuit of understanding the variance in ionic conductivity observed among lithium ternary chlorides with similar structures, we conducted an in‐depth analysis. We examined the ionic radii of Sm, Er, and In (Sm^3+^: 96 pm, Er^3+^: 89 pm, In^3+^: 80 pm) and their influence on the lattice parameters of LMC, resulting in extended M─Cl and Li─Cl bond lengths within their respective polyhedra, as shown in Figure S15 (Supporting Information). This elongation of Li─Cl bonds tends to reduce the electrostatic attraction between Li⁺ and Cl⁻ ions, facilitating easier migration of alkali ions and subsequently increasing the ionic conductivity of these materials.^[^ ^32^ ^]^

**Figure 4:**
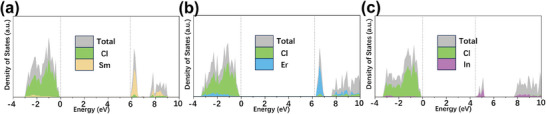
HSE06‐derived DOS for a) LSC, b) LEC, and c) LIC.

In addition to exceptional Li‐ion conducting properties, our first‐principles calculations also verified wide electrochemical windows, low electronic conductivity, and favorable interface compatibility with electrodes in these chloride materials (see the Supporting Information). As depicted in **Figure** **4**a–c, we calculated the element‐resolved PDOS of LSC, LEC, and LIC using HSE06 functional. The results show that LSC, LEC, and LIC possess substantial band gap values of 5.88, 6.20, and 4.44 eV, respectively. The insulating nature with large bandgap values of these chloride materials indicates they are highly unfavorable for electronic conduction, which is crucial for SSEs. Simultaneously, the PDOS results reveal that the Cl^−^ and M^3+^ (M = Sm, Er, and In) orbital components dominate the valence band maximum (VBM) and conduction band minimum (CBM), respectively, indicating that they will be the initial species to undergo oxidized and reduced at high and low voltages, respectively. To determine the thermodynamic intrinsic electrochemical windows for LSC, we calculated the equilibrium voltage profile and corresponding phase equilibria as functions of applied potential, with reference to Li^+^/Li, using the methodology aligned with the Materials Project database. These three chloride materials all show wide electrochemical windows with anodic limits of 4.30, 4.33, and 4.38 V (shown in Figure S16, Supporting Information), respectively, and cathodic limits of 0.74, 0.70, and 2.55 V, respectively. These thermodynamic intrinsic windows are significantly wider than many current sulfide and oxide SSEs such as LGPS (1.72−2.29 V), Li_3_PS_4_ (1.71−2.31 V), LISICON (1.44−3.39 V), and Li_0.33_La_0.56_TiO_3_ (1.75−3.71 V).^[^ ^33^, ^34^ ^]^ Despite having lower reduction limits than many other SSEs, LSC, LEC, and LIC are not thermodynamically stable against Li metal, with projected decomposition energies of 0.20−0.81 eV per atom when in equilibrium with Li. Our results indicate that the *P*3_1_12 LSC, LEC and LIC are 0.7, 12.3 and 12.7 meV lower in formation energies, respectively, compared to the most stable phases in the Material Project database. Although the electrochemical stabilities are improved, as the LMC possesses a negative Δ*E*_above_hull_ (where a negative value indicates a lower energy relative to the previously established convex hull). The electrochemical windows of LMC are similar to the phases that have been reported, because the enhancements in stability ascertained using thermodynamic criteria are not significant.

The decomposition energies of LMC (M = Sm, Er, In) by reduction and oxidation reactions as a function of the chemical potential of lithium are shown in Figure S17 (Supporting Information). The decomposition energies show a consistent trend across the M^3+^ cations. The consistency can be attributed to the uniform oxidation products, i.e., MCl_3_ and Cl_2_. However, a distinct dependence was observed regarding the decomposition energies of the reduction reaction based on the elemental block of cation M^3+^. Particularly, the decomposition energy for the reduction reaction of LIC is four times higher compared to that of LSC and LEC when assessed against the Li anode (at 0 V), suggesting that LIC is unsuitable on the anode side. Detailed reduction and oxidation reactions of SSEs as a function of the chemical potential of Li in Figure S17 (Supporting Information) are listed in Table S2 (Supporting Information).

Next, the chemical stability of LMC in relation to cathode materials was assessed by calculating the maximum decomposition energy from potential chemical interactions between LMC and the cathode materials. As shown in Figure S18 (Supporting Information), a heat map presents the calculated reaction energies between LMC and various cathode materials, namely LFP, LMO, NCM, and LCO. The reaction energy between the sulfides (Li_3_PS_4_, LGPS, and Li_6_PS_5_Cl) and four cathode materials is also included in Figure S18 (Supporting Information) to compare the chemical stabilities of the chlorides and sulfides. The decomposition energies for the chemical reaction in Figure S18 (Supporting Information), detailed chemical reactions, and decomposition phases between cathode materials and lithium chlorides/sulfides are listed in Table S3 (Supporting Information). In general, LMC exhibits lower decomposition energies against LFP than against LMO and LCO, while LMC shows the highest decomposition energy against NCM. The average decomposition energies of LMC against LMO, LCO, LFP, and NCM are approximately 8, 36, 41, and 57 meV per atom, respectively. In contrast, the average decomposition energies of sulfides against LFP, LMO, LCO, and NCM are approximately 107, 314, 353, and 372 meV per atom, respectively. These values for sulfides are significantly higher than those for chlorides, underscoring the superior chemical stability of chlorides compared to sulfides. Although the decomposition energies indicate that chemical reactions between LMC and cathode materials are thermodynamically favorable, kinetic barriers may still inhibit these reactions. Moreover, decomposition phases at the interface, due to the low reaction energies of the chlorides, may inhibit further decomposition of the primary phase, which ensures the interface stability of LMC.

Before the conclusion, it is worth noting that several studies have reported that halide solid electrolytes synthesized by the ball‐milling method often exhibit higher ionic conductivity compared to those synthesized by annealing.^[^ ^20^ ^]^ Therefore, we recommend the ball‐milling method for the synthesis of Li_3_SmCl_6_ using LiCl and SmCl_3_ as precursors.

## 3 Conclusion

In summary, through crystal structure prediction and first‐principles calculations, we have identified Li_3_SmCl_6_ as a novel chloride superionic conductor. Exhibiting *P*3_1_12 space group symmetry, it demonstrates a high ionic conductivity of 15.46 mS cm^−1^ at room temperature. Our structural analysis reveals that this remarkable conductivity is largely attributed to its unique in‐plane cation ordering and stacking sequences, facilitating superior Li^+^ transport properties. Additionally, Li_3_SmCl_6_ has a broad electrochemical stability range of 0.734.30 V, exhibiting exceptional chemical interface stability when utilized in conjunction with high‐voltage cathodes. Notably, the Sm component in the Li_3_SmCl_6_ structure can be substituted with metal elements Er and In, which also exhibit characteristics of promising performance as chloride solid‐state electrolytes. This research underscores the potential of structure searching in uncovering novel solid‐state electrolytes and provides valuable insights for laboratory synthesis.

## 4 Experimental Section

The structure of LSC was predicted and confirmed by the crystal structure analysis by particle swarm optimization (CALYPSO) code, which was successfully predicted the structures of diverse lithium battery systems.^[^ ^35^, ^36^, ^37^ ^]^ A search of 30 generations with 50 structures in each generation was conducted. Particularly, 20 structures were generated using the particle swarm optimization algorithm among the 50 structures in each generation, while the remaining 30 structures were randomly generated according to the point group symmetry. This sampling methodology guarantees both the structural heritability and the diversity of structures. Then, among the resulting structures, the top 20 structures with the lowest energy were selected as candidates for the ground‐state structure. Those structures are further optimized to identify the lowest‐energy structure.

All the first‐principles calculations were performed using the Vienna Ab initio Simulation Package (VASP).^[^ ^38^ ^]^ The generalized gradient approximation (GGA) of Perdew−Burke−Ernzerhof (PBE) form and the projector‐augmented wave (PAW) pseudopotentials were employed to describe the exchange−correlation functions and election‐ion interactions.^[^ ^39^ ^]^ The kinetic energy cutoff of 520 eV was used for the plane‐wave basis set. The convergence criteria for total energy and Hellmann‐ Feynman force were set to 1.0 × 10^−6^ eV and 1.0 × 10^−2^ eV Å^−1^, respectively. The Monkhorst‐Pack *k*‐points sampling was employed for the Brillouin zone integration: 4 × 4 × 1 for relaxation, and 9 × 9 × 2 for self‐consistent calculation. The validity of bandgap and projected density of states (PDOS) were further confirmed by the calculations using the Heyd‐Scuseia‐Ernzerhof functional (HSE06) within DS‐PAW software.^[^ ^40^, ^41^ ^]^ Density functional perturbation theory was used to calculate the Hessian matrices and phonon frequency, using a 2 × 2 × 1 supercell with 3 × 3 × 2 *k*‐mesh. Phonopy was used to obtain second‐order force constant matrices, thermal properties, and phonon dispersion curves.^[^ ^42^ ^]^

The ab initio molecular dynamics (AIMD) simulations were carried out utilizing a 2 × 2 × 1 supercell within the canonical (NVT) ensemble by means of Nosé‐Hoover thermostat, as implemented in the VASP code. A 1 × 1 × 1 *k*‐points grid was adopted. Li_3_MCl_6_ (LMC, M = Sm, Er, In) structures were first statically relaxed and then heated from an initial temperature of 100 K to the target temperature (600–1000 K) by velocity scaling over a time period of 2 ps, and then equilibrated at the desired temperature for 10 ps in the NVT ensemble using a Nosé–Hoover thermostat. And then to sample an adequate number of diffusion events for minimization of statistical variances in calculated diffusivity, AIMD simulations were performed for a total time of 60–130 ps at varying equilibrium temperature. A total mean‐squared displacement (TMSD) of 3200–8600 Å2 was reached, resulting in an estimated relative standard deviation of the fitted diffusivity in the acceptable range of 16–24%. The post‐processing analysis of AIMD trajectory was performed using the Python Materials Genomics (Pymatgen) package.^[^ ^43^, ^44^ ^]^ More details about Li^+^ diffusivity/conductivity are provided in the Supporting Information. The migration pathway and activation energy barrier of Li^+^ were verified through the utilization of the bond valence site energy method (BVEM).^[^ ^45^, ^46^, ^47^ ^]^ The entire workflow of this study is illustrated in Figure S1.

## Conflict of Interest

The authors declare no conflict of interest.

## Supporting information

Supporting Information

## Data Availability

The data that support the findings of this study are available from the corresponding author upon reasonable request.
