# Supplementary material for: Prediction of Novel Trigonal Chloride Superionic Conductors as Promising Solid Electrolytes for All‐Solid‐State Lithium Batteries
Source: Adv Sci (Weinh). 2024 Jul 9;11(34):2404213. doi: 10.1002/advs.202404213 (PMC11425969; doi:10.1002/advs.202404213)
Supplement: Supplementary file 1 — Supporting Information [file ADVS-11-2404213-s001.docx]

Supporting Information

Prediction of novel trigonal chloride superionic conductors as promising solid electrolytes for all-solid-state lithium batteries

Yao Wang,^#^ Ziang Ren,^#^ Jinsen Zhang, Shaohua Lu, Chenqiang Hua, Huadong Yuan, Jianmin Luo, Yujing Liu, Jianwei Nai, Xinyong Tao *


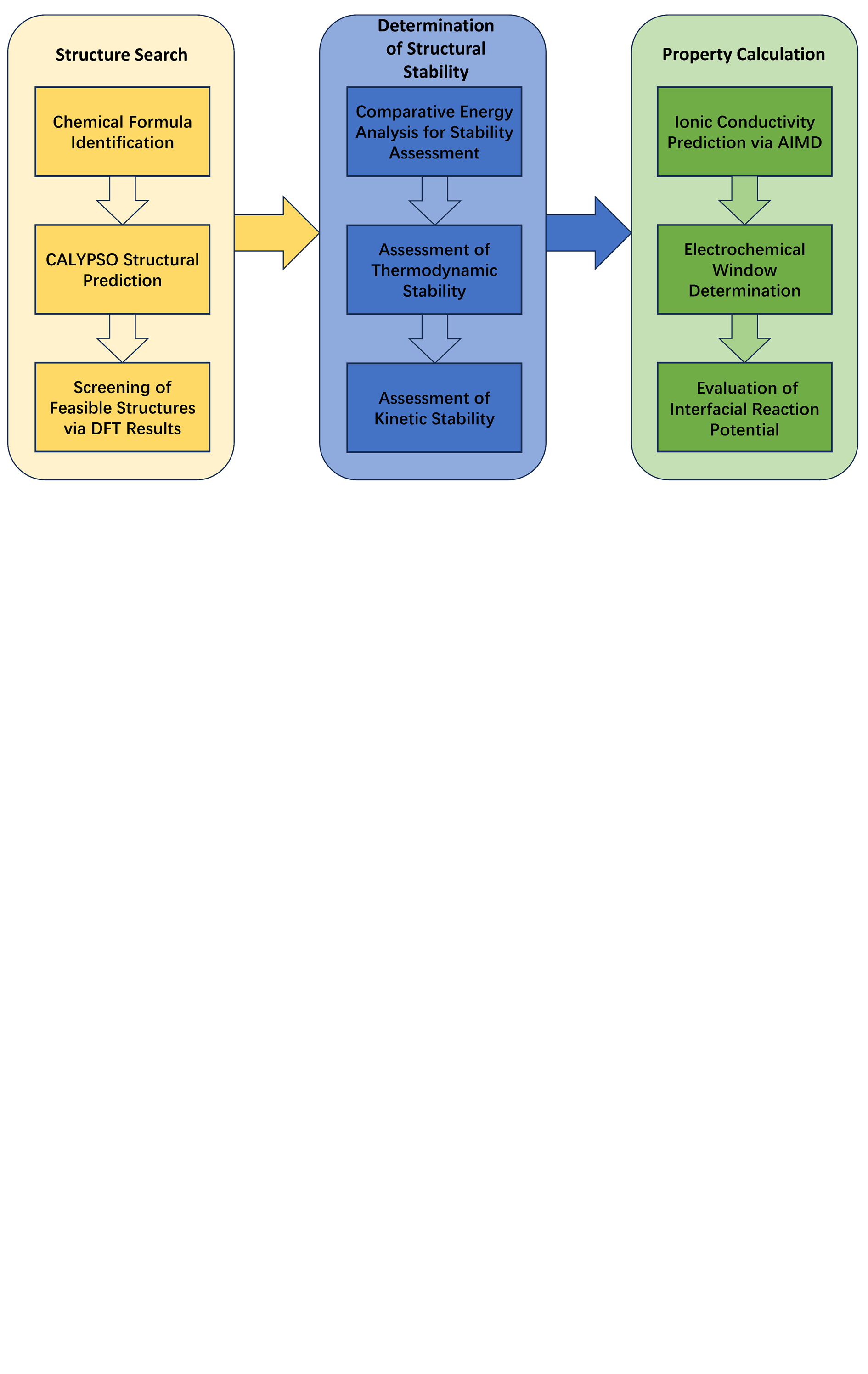

**Figure S1.** Flowchart of the CALYPSO structural search to SSEs performance evaluation process.


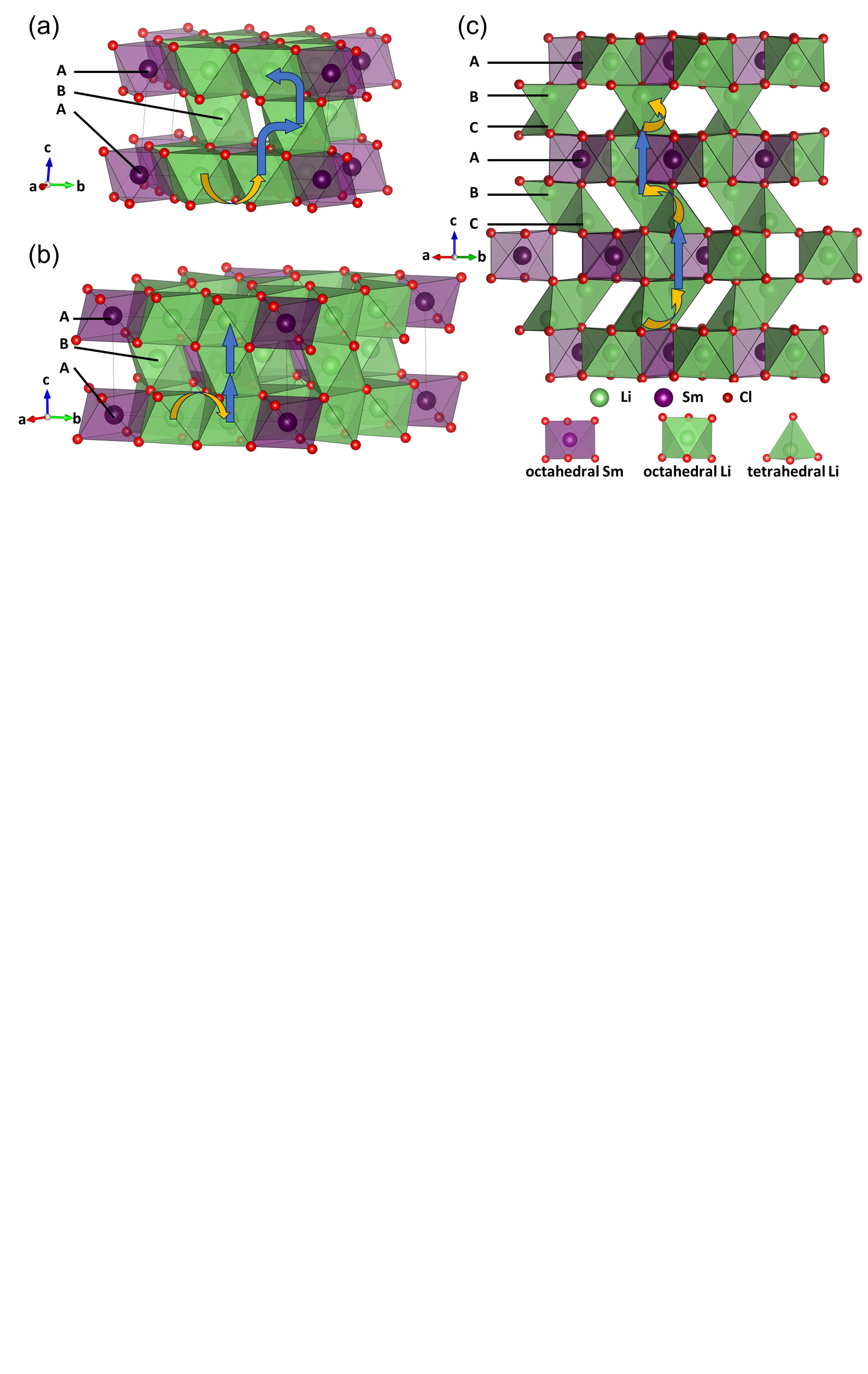


**Figure S2.** Structures of LSC with (a) *C*2/*m*, (b) *Pm*1, and (c) *P*3_1_12 (this work) space group


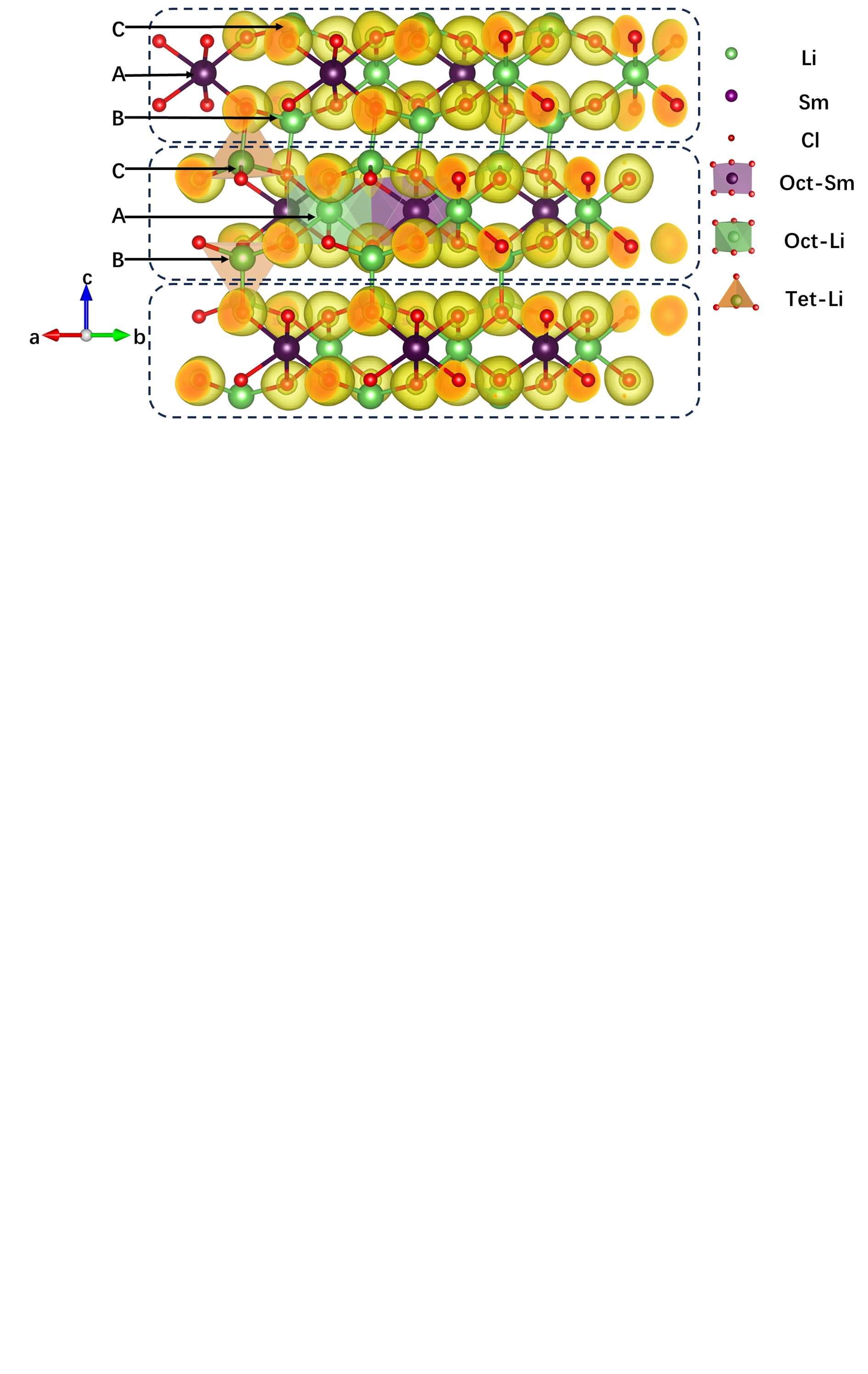


**Figure S3.** 3D Electron Localization Function (ELF) map of LSC with a threshold of 0.8 a.u.


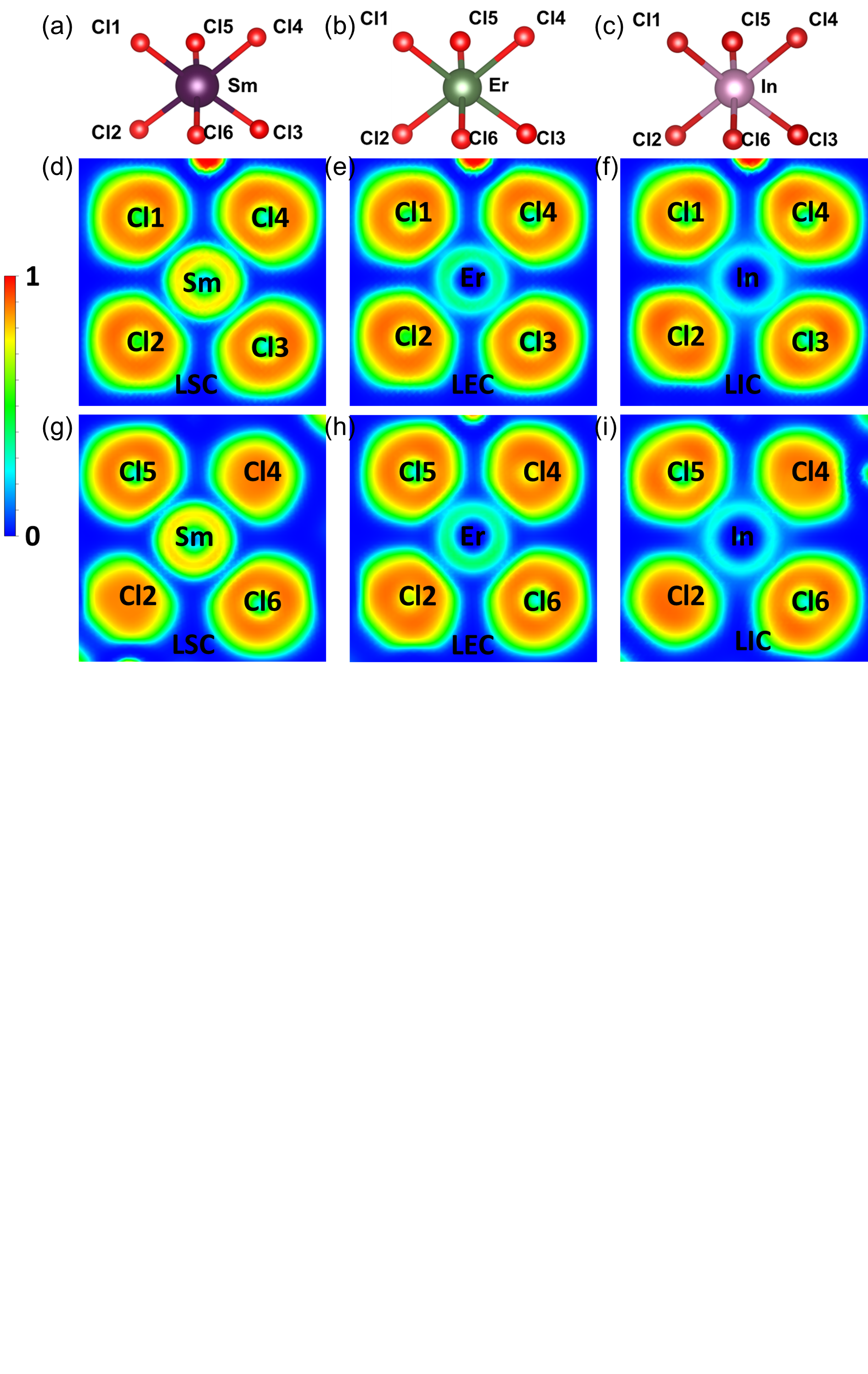


**Figure S4.** (a-c) Molecular structure models of MCl_6_ octahedra. (d-i) 2D electron localization function (ELF) map of LMC.


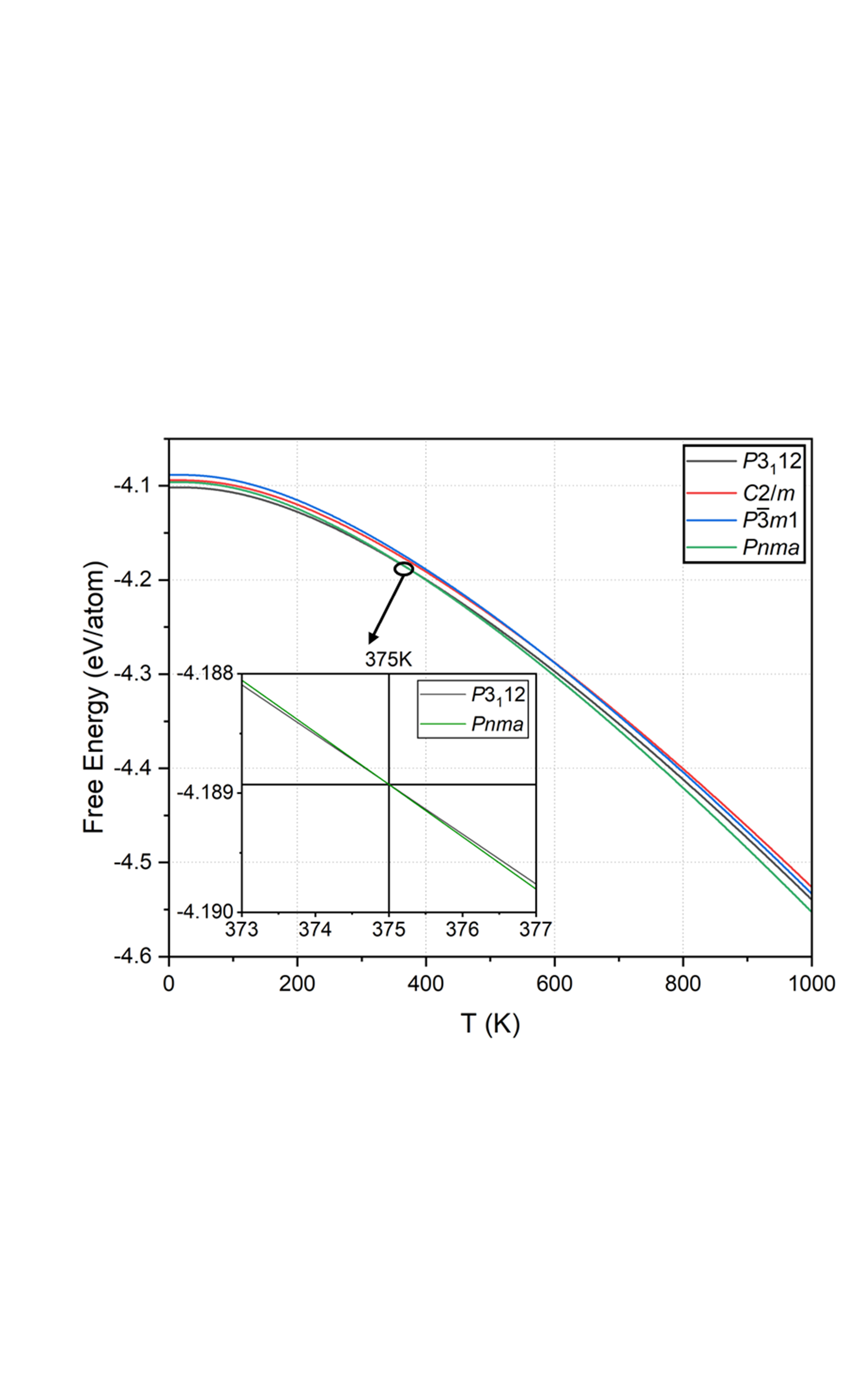


**Figure S5.** Comparison of free energy for structures of LSC with different space groups as a function of temperature.


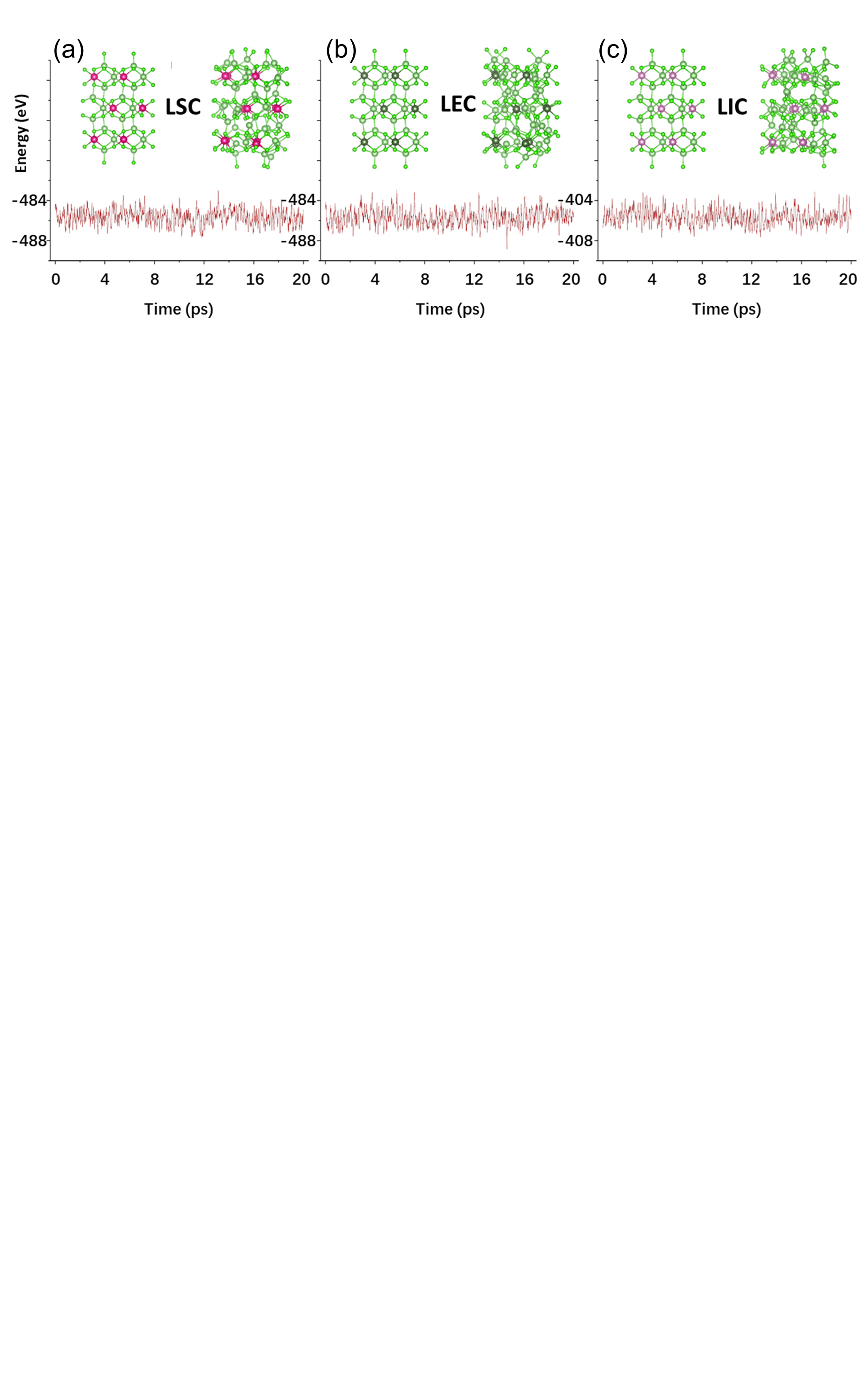


**Figure S6.** Total energy fluctuation during the AIMD simulations at 600 K of (a) LSC, (b) LEC, and (c) LIC.


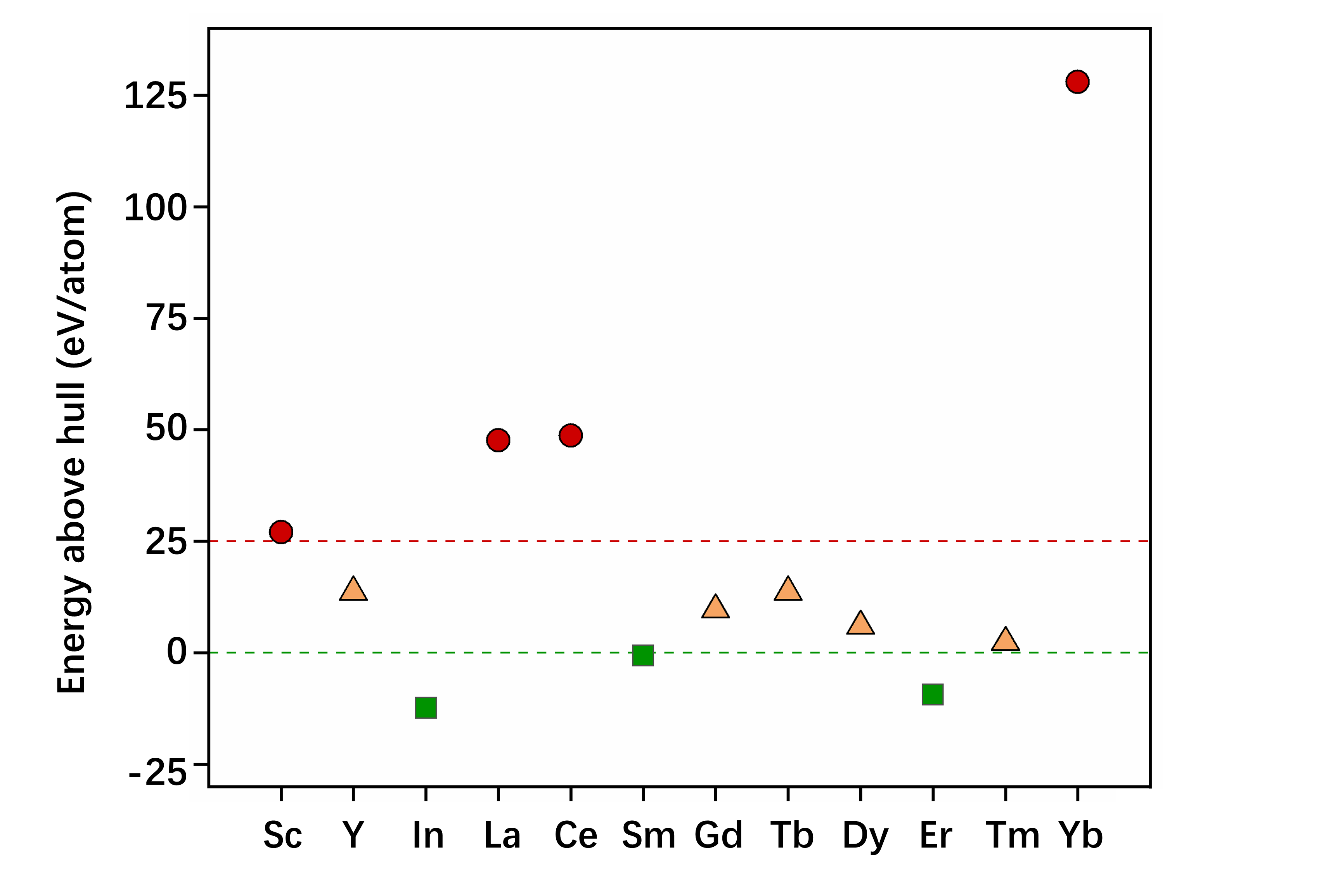


**Figure S7.** The calculated energy above hull (Δ*E*_above_hull_) of LMC, where M = Sc, Y, In, La, Ce, Sm, Gd, Tb, Dy, Er, Tm and Yb.


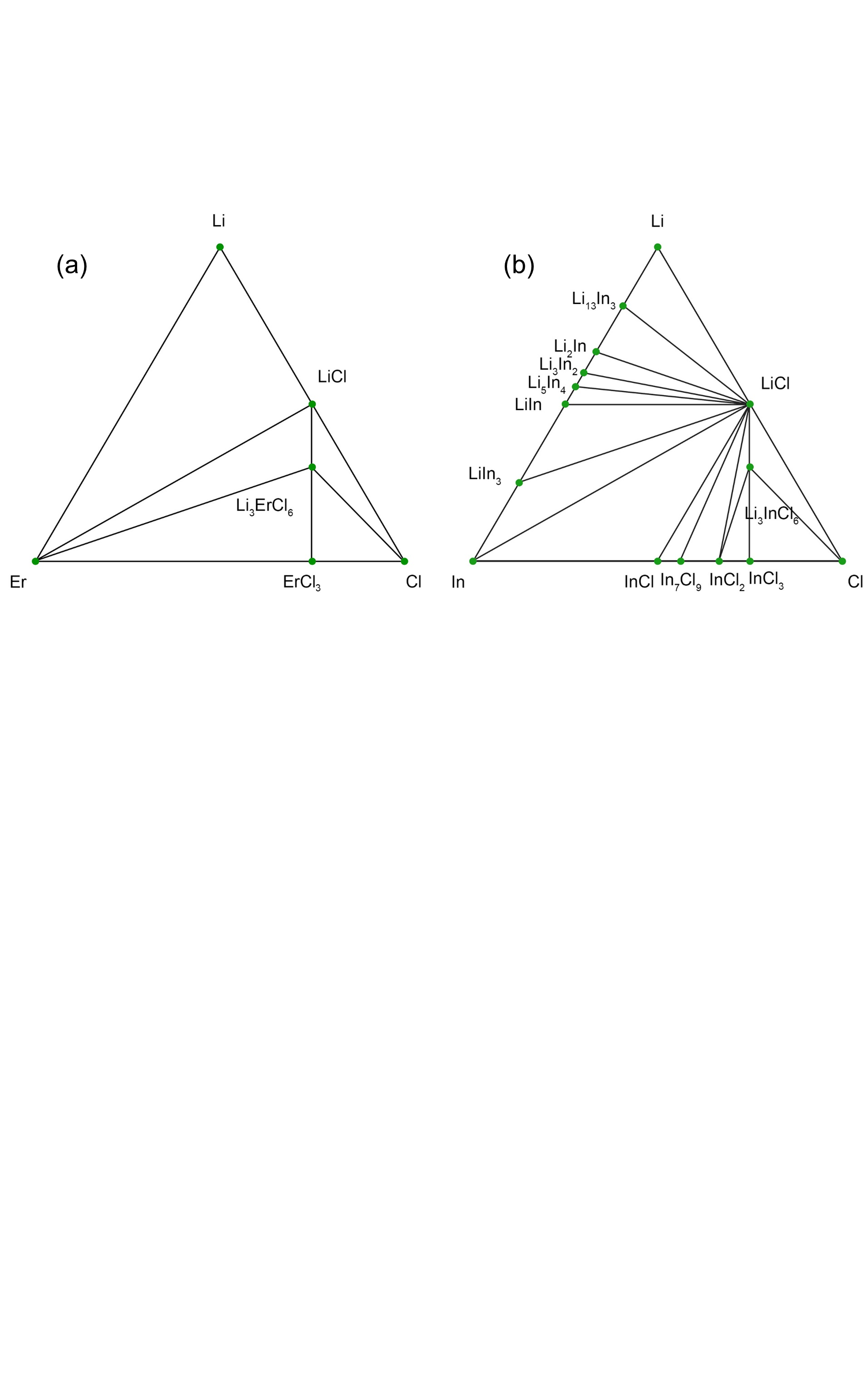


**Figure S8.** Phase diagram of (a) LEC and (b) LIC.


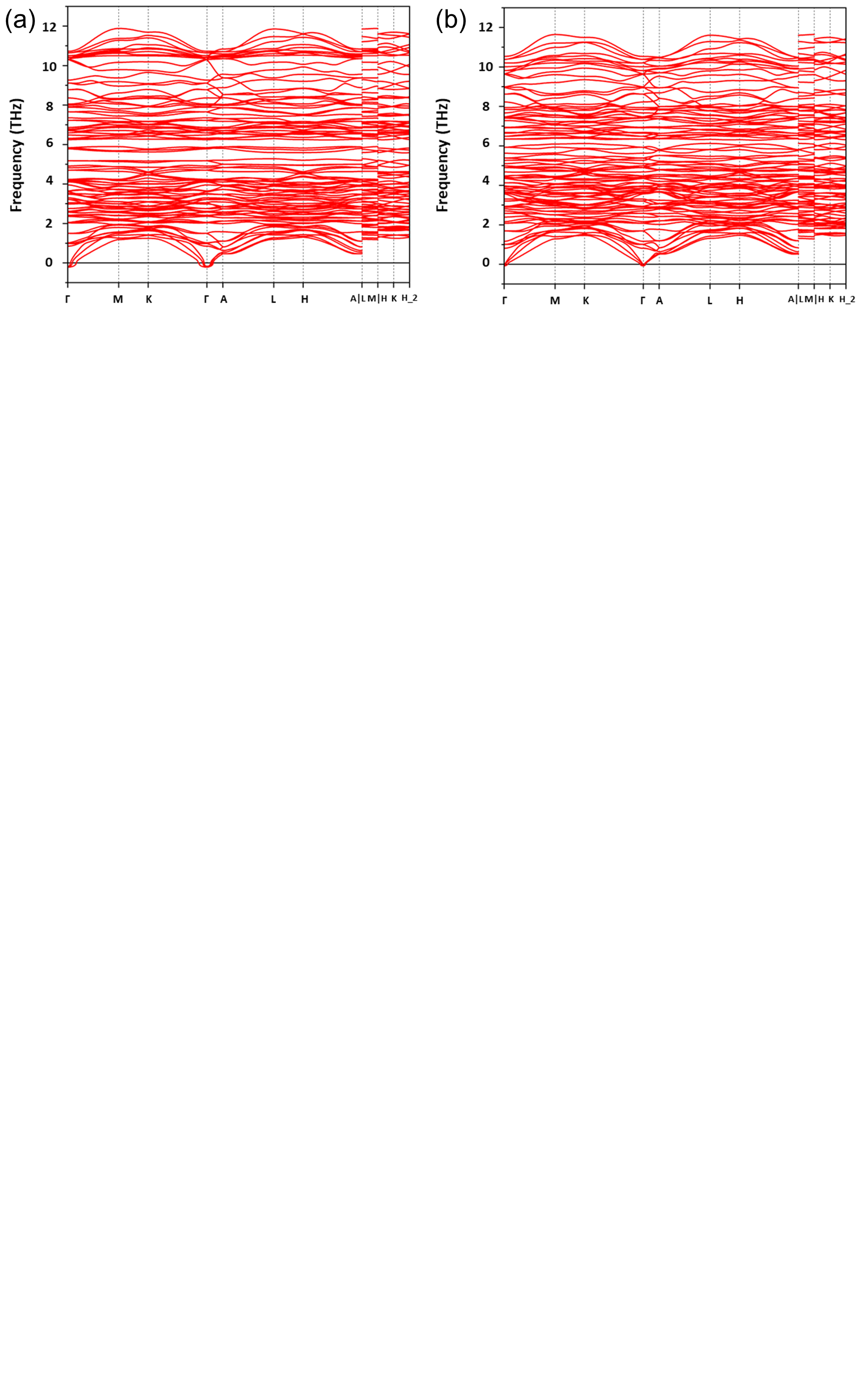


**Figure S9.** The phonon spectra of (a) LEC, and (b) LIC


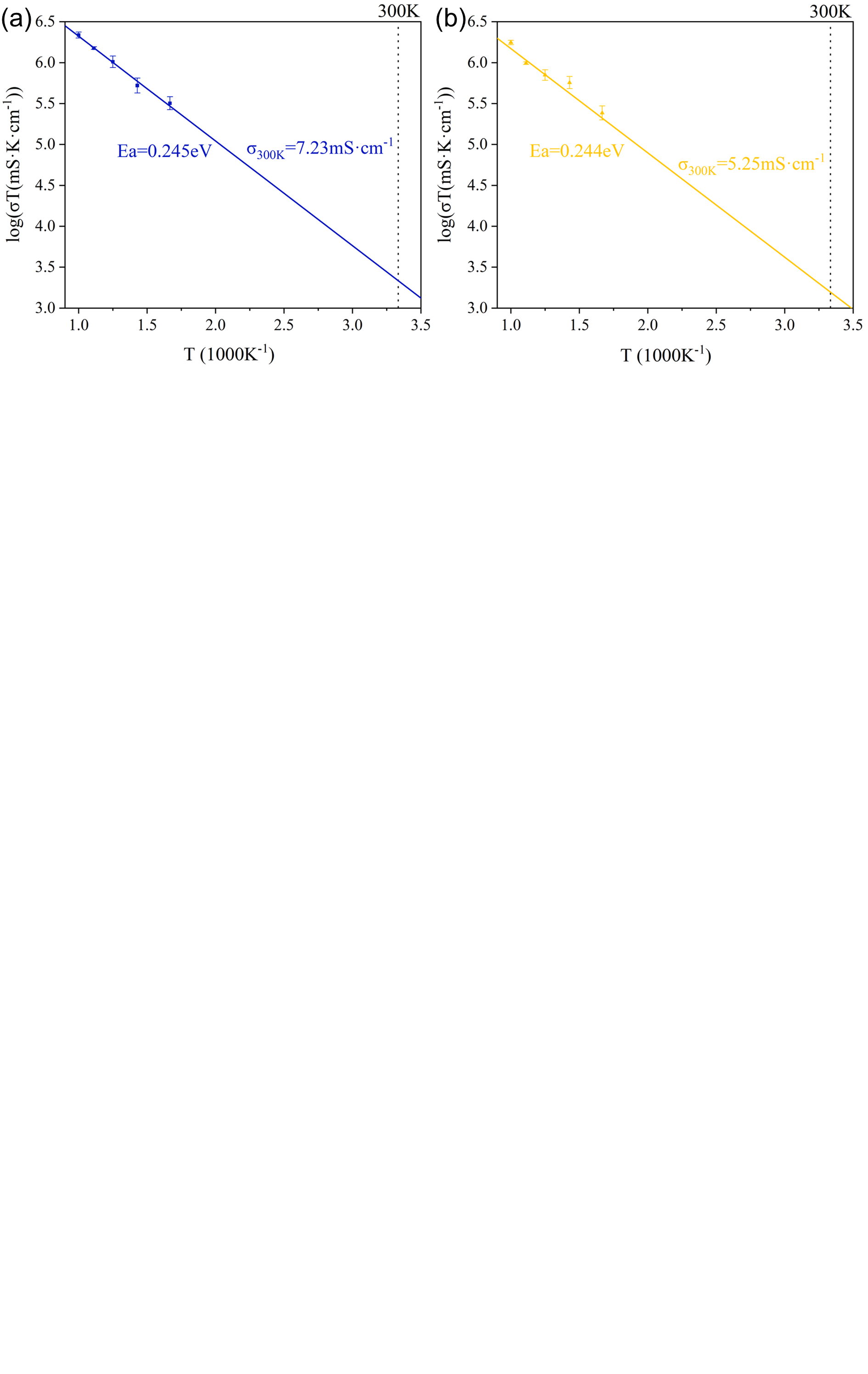


**Figure S10.** Arrhenius plots of Li^+^ diffusivity for (a) LEC and (b) LIC from AIMD simulations.


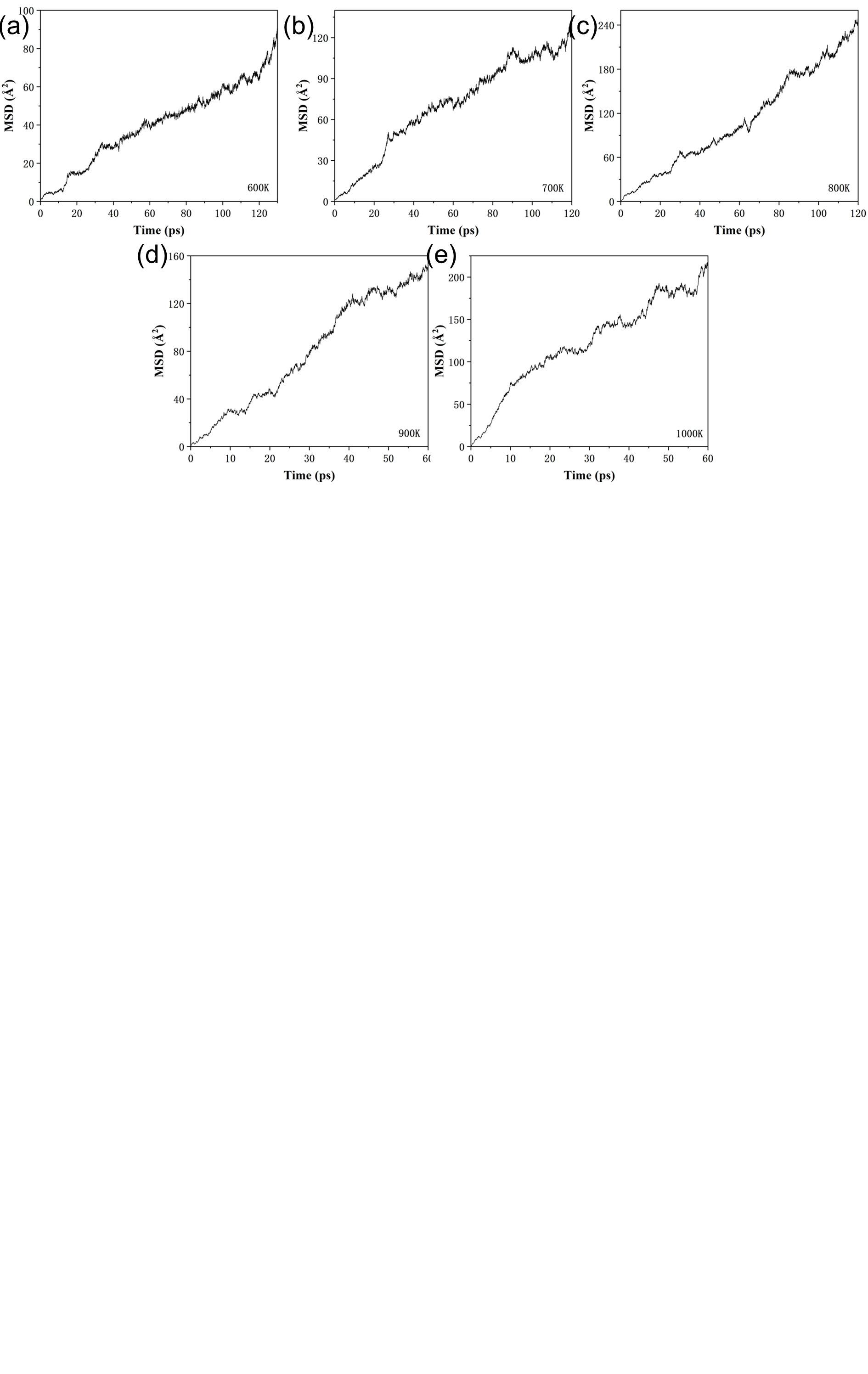


**Figure S11.** MSD plots of LSC for AIMD simulations. (a) 130ps AIMD simulation at 600K. (b) 120ps AIMD simulation at 600K. (c) 120ps AIMD simulation at 800K. (d) 60ps AIMD simulation at 900K. (e) 60ps AIMD simulation at 1000K.


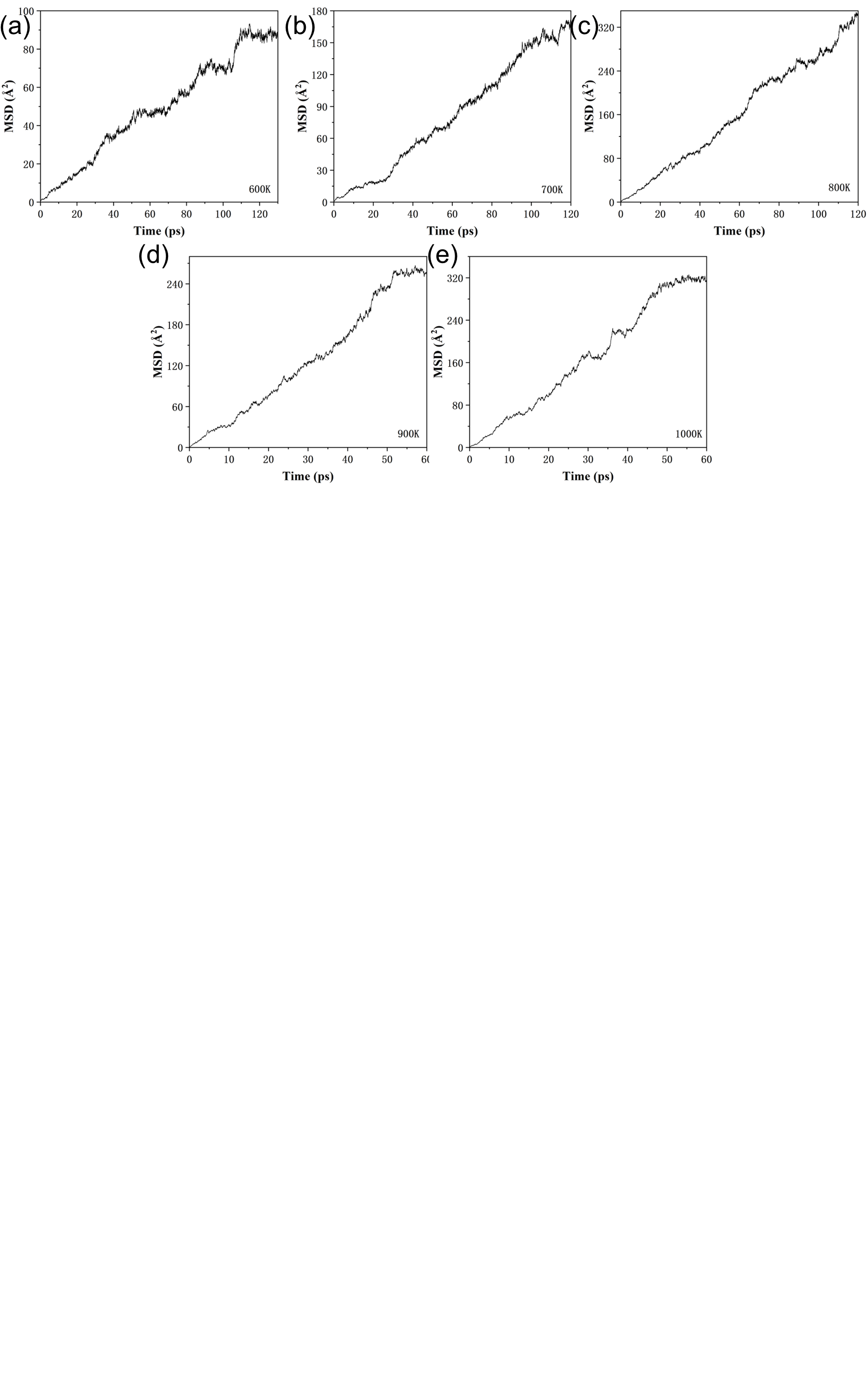


**Figure S12.** MSD plots of LEC for AIMD simulations. (a) 130ps AIMD simulation at 600K. (b) 120ps AIMD simulation at 600K. (c) 120ps AIMD simulation at 800K. (d) 60ps AIMD simulation at 900K. (e) 60ps AIMD simulation at 1000K.


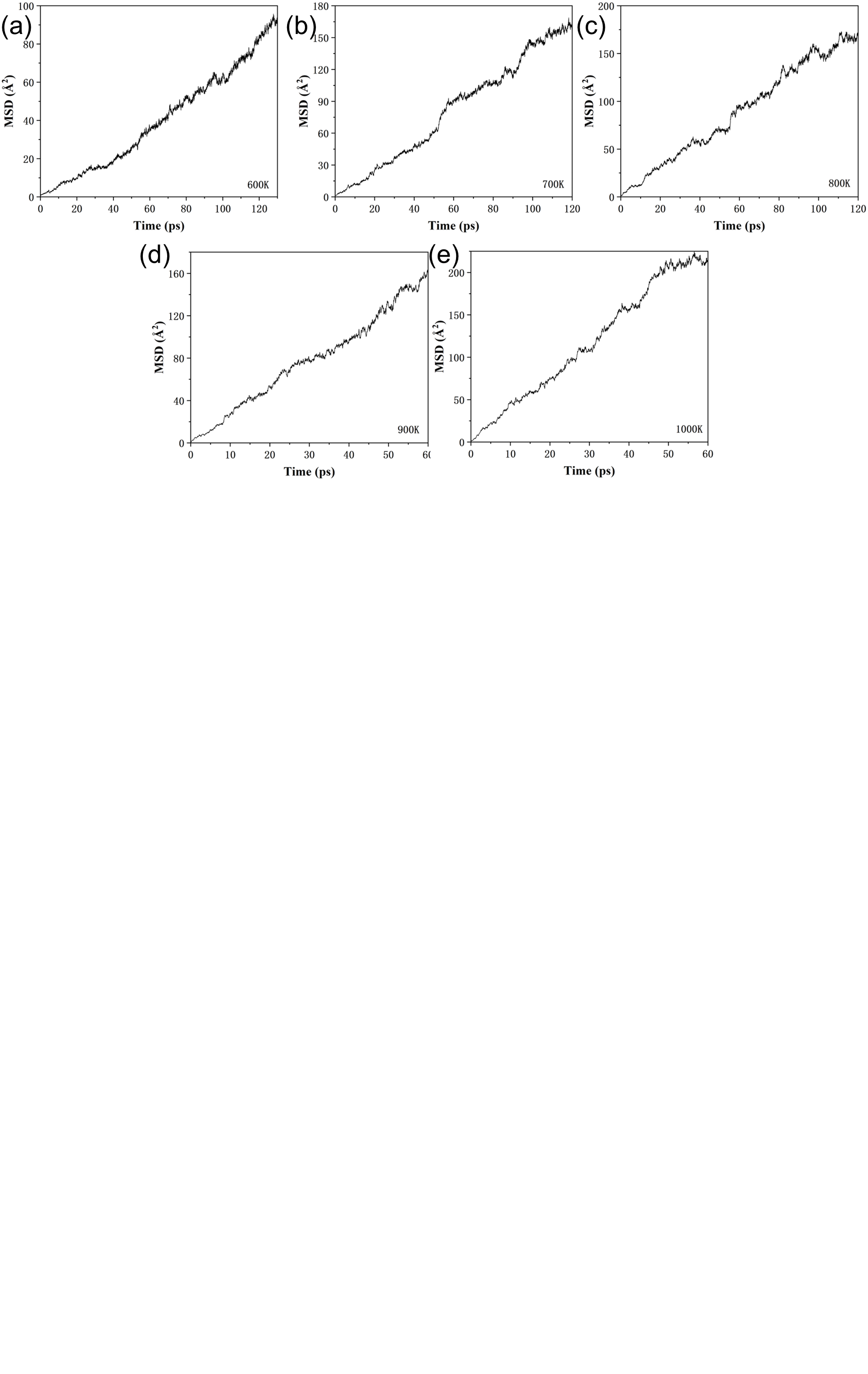


**Figure S13.** MSD plots of LIC for AIMD simulations. (a) 130ps AIMD simulation at 600K. (b) 120ps AIMD simulation at 600K. (c) 120ps AIMD simulation at 800K. (d) 60ps AIMD simulation at 900K. (e) 60ps AIMD simulation at 1000K.


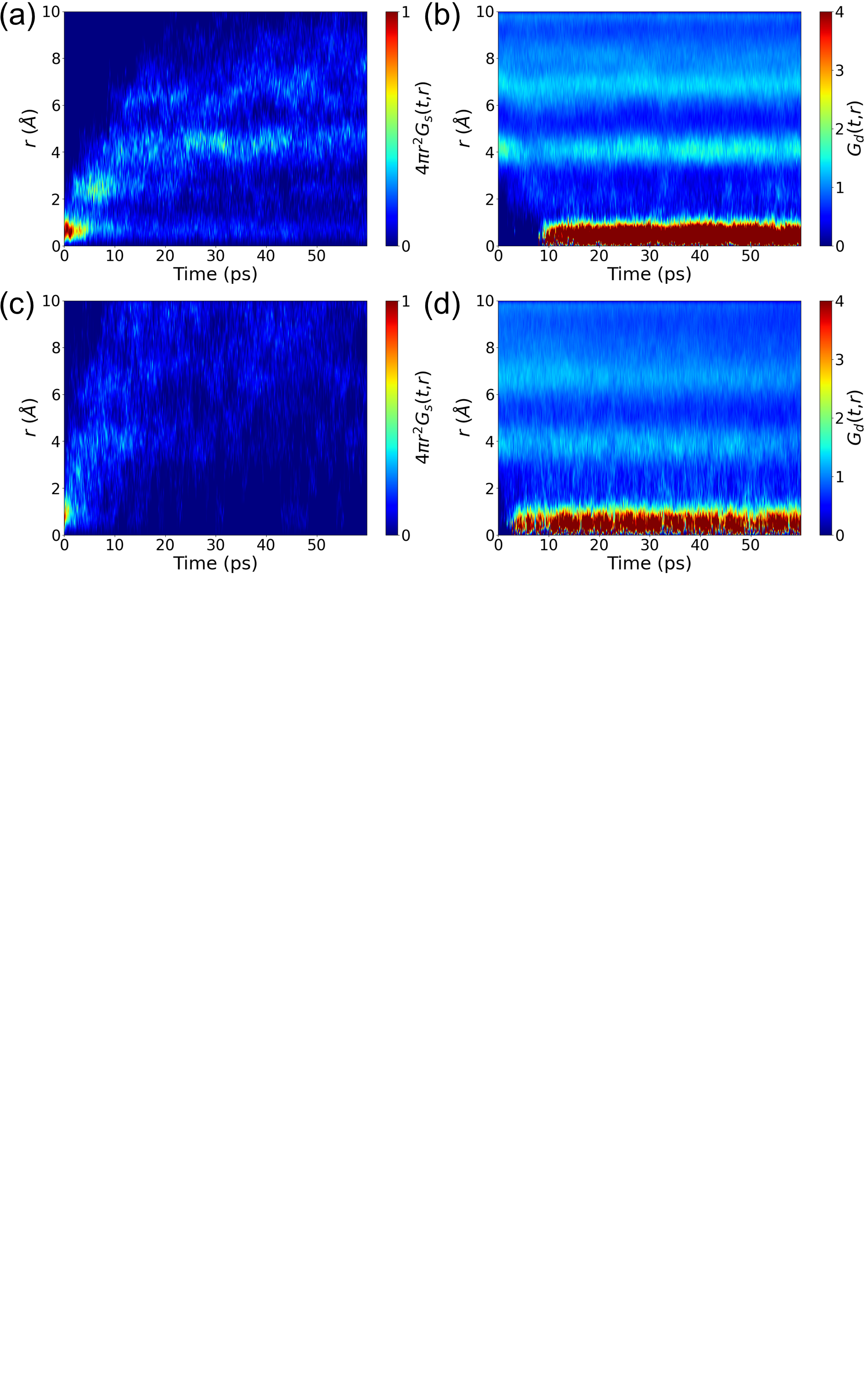


**Figure S14.** Van Hove correlation functions of LSC Li^+^ obtained from AIMD simulations at (a-b) 600 K and (c-d) 1000 K.


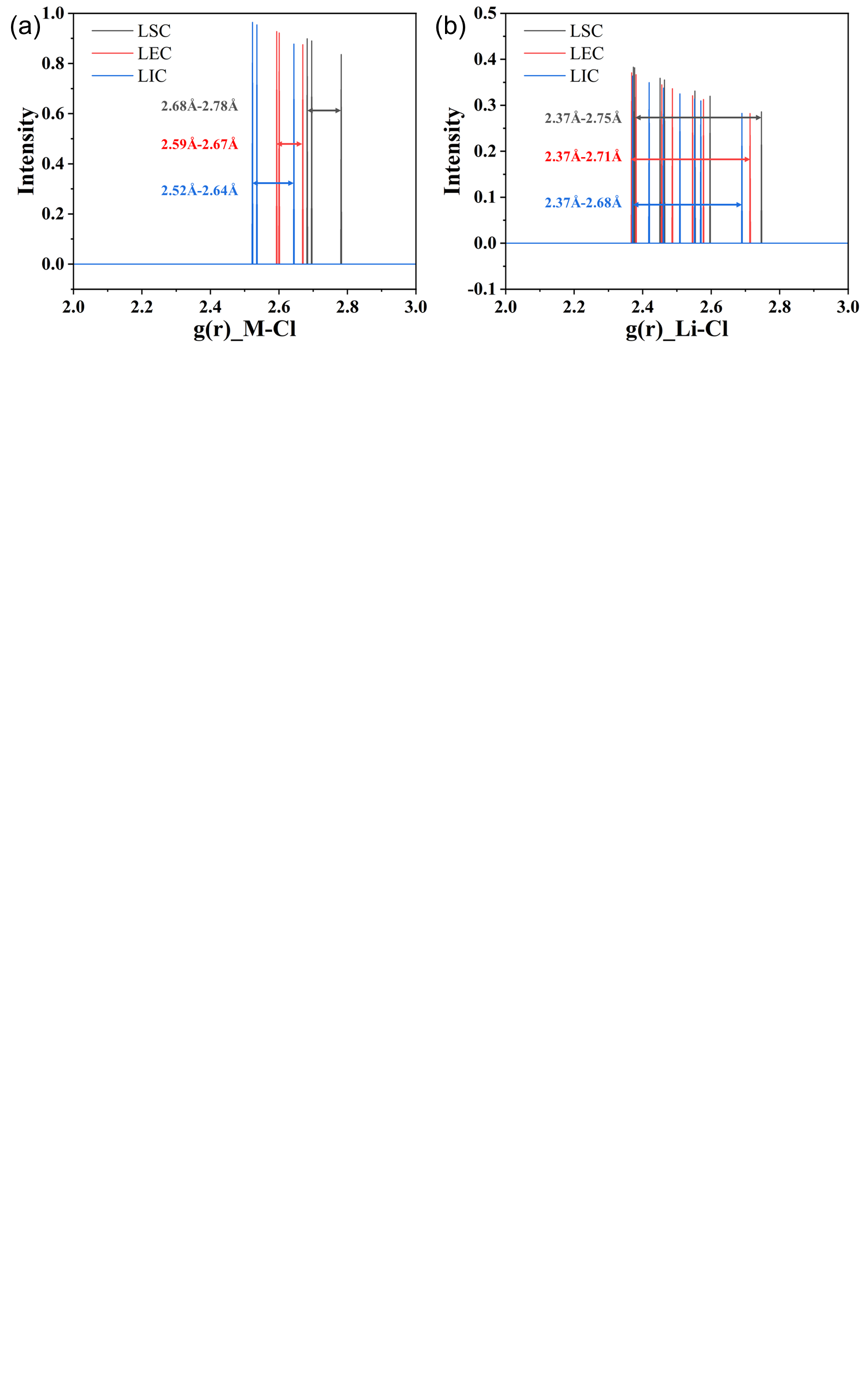


**Figure S15.** (a) Pair correlation functions (PCFs) of LMC (M = Sm, Er, In) for M-Cl. (b) Pair correlation functions (PCFs) of LMC for Li-Cl.


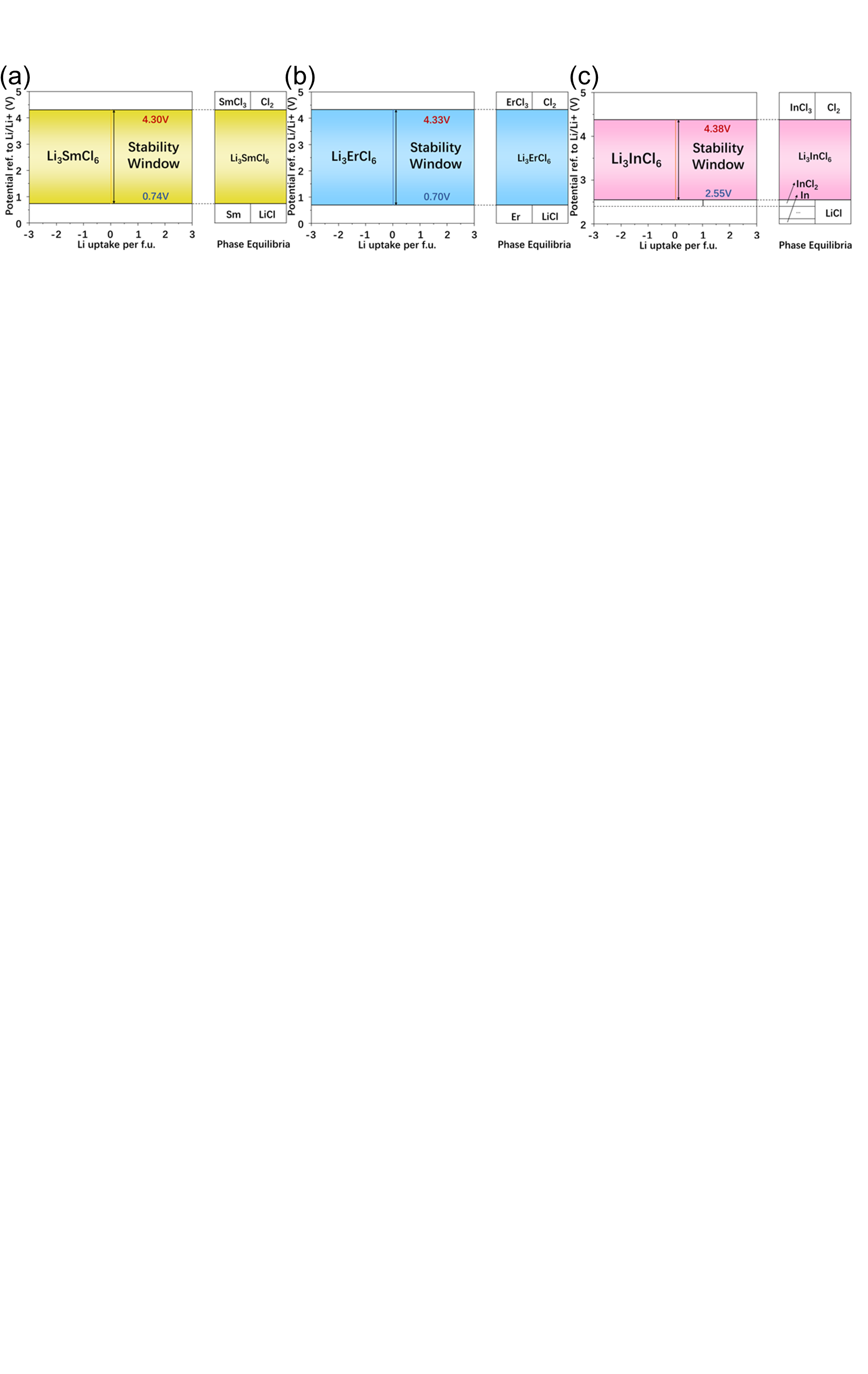


**Figure S16.** Thermodynamic profiles and phase equilibria for (a) LSC, (b) LEC, and (c) LIC.


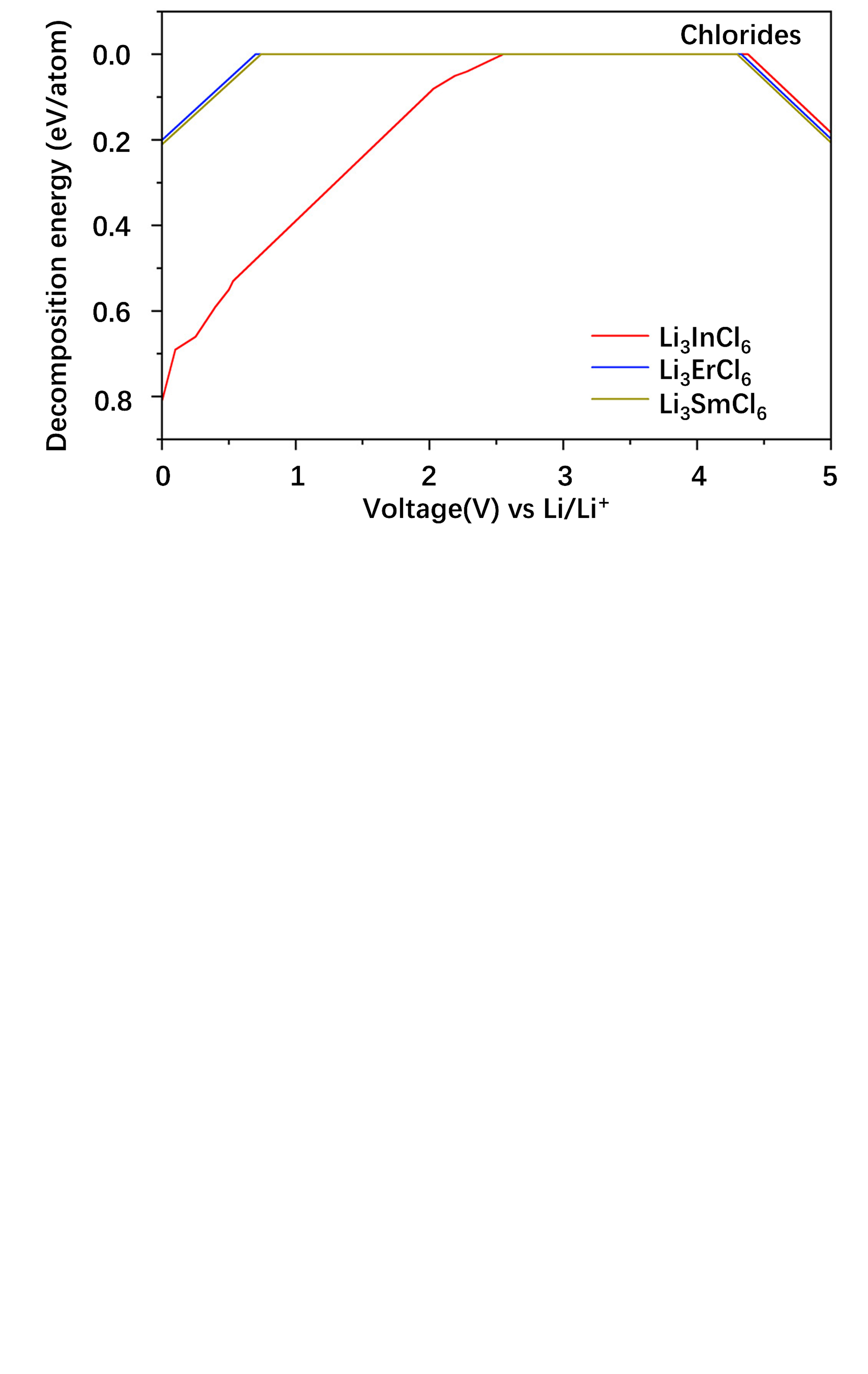


**Figure S17.** The decomposition energy of LMC as a function of the chemical potential of lithium.


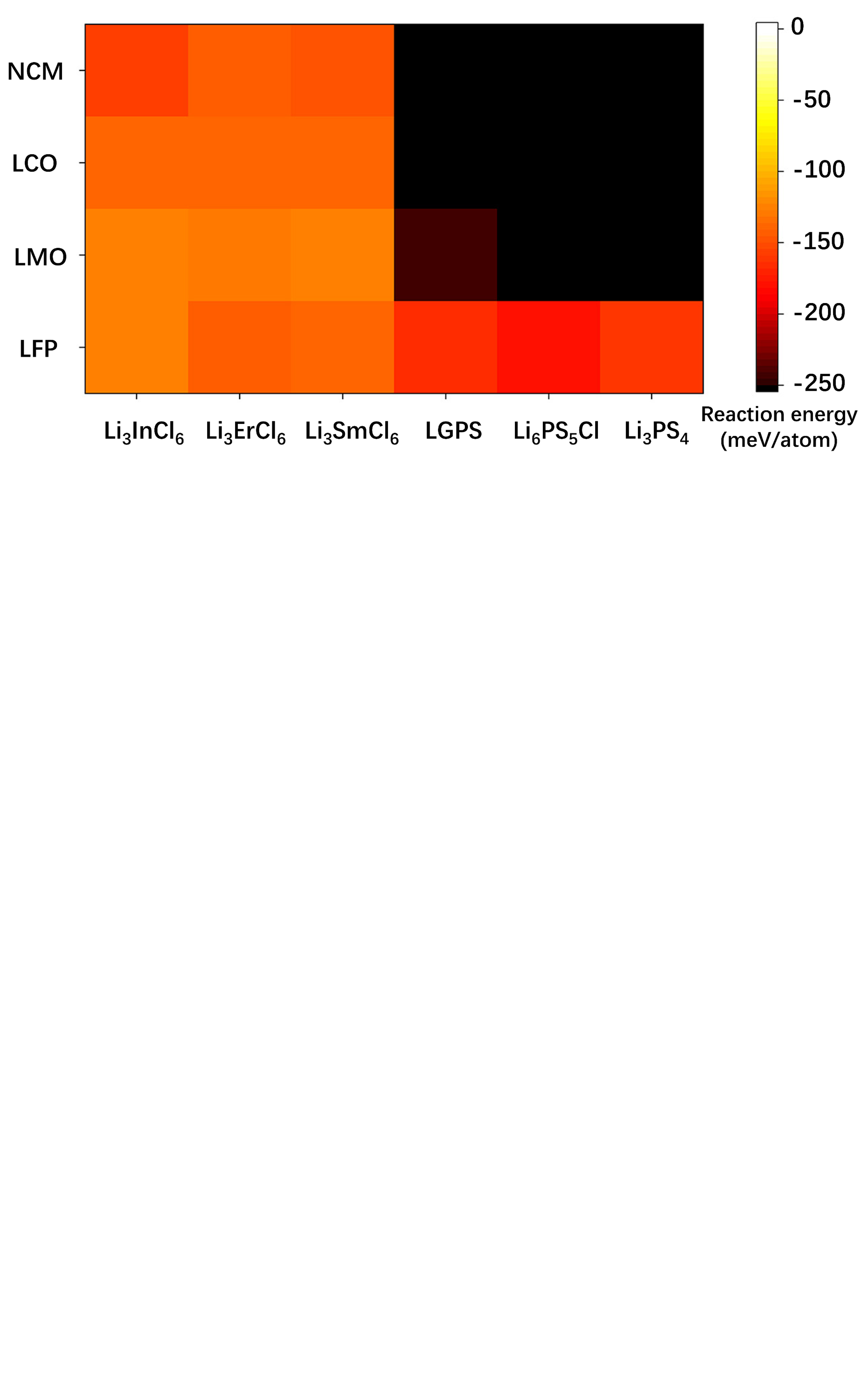


**Figure S18.** Heat map of the reaction energy between lithium chlorides, sulfides, and four cathode materials.

**Table S1** Structure parameters of Li_3_SmCl_6_.

Space group: *P*3_1_12

a = b = 6.7376 Å, c = 18.6127 Å.

| Atom | Wyckoff position | Atomic coordinates | | |
| --- | --- | --- | --- | --- |
|  |  | x | y | z |
| Sm | 3a | 0.88076 | 0.11924 | 0.33333 |
| Cl1 | 6c | 0.20429 | 0.73311 | 0.41046 |
| Cl2 | 6c | 0.89162 | 0.10264 | 0.41072 |
| Cl3 | 6c | 0.57273 | 0.45681 | 0.08686 |
| Li1 | 3a | 0.21187 | 0.78813 | 0.33333 |
| Li2 | 6c | 0.53027 | 0.07058 | 0.21824 |

**Table S2** Reduction and oxidation reactions of Li_3_MCl_6_ (M= In, Er, and Sm) as a function of the chemical potential of lithium.

| Solid electrolyte | Potential (V) | E_D_  (eV/atom) | Phase equilibria |
| --- | --- | --- | --- |
| Li_3_InCl_6_ | 0.00 | 0.81 | Li_13_In_3_, LiCl |
|  | 0.10 | 0.69 | Li_2_In, LiCl |
|  | 0.25 | 0.66 | Li_3_In_2_, LiCl |
|  | 0.40 | 0.59 | Li_5_In_4_, LiCl |
|  | 0.50 | 0.55 | LiIn, LiCl |
|  | 0.50 | 0.55 | LiIn_3_, LiCl |
|  | 0.53 | 0.53 | In, LiCl |
|  | 2.03 | 0.08 | InCl, LiCl |
|  | 2.19 | 0.05 | In_7_Cl_9_, LiCl |
|  | 2.28 | 0.04 | InCl_2_, LiCl |
|  | 2.55 | 0.00 | Li_3_InCl_6_ |
|  | 4.38 | 0.00 | InCl_3_, Cl_2_ |
| Li_3_ErCl_6_ | 0.00 | 0.20 | Er, LiCl |
|  | 0.70 | 0.00 | Li_3_ErCl_6_ |
|  | 4.33 | 0.00 | ErCl_3_, Cl_2_ |
| Li_3_SmCl_6_ | 0.00 | 0.21 | Sm, LiCl |
|  | 0.74 | 0.00 | Li_3_SmCl_6_ |
|  | 4.30 | 0.00 | SmCl_3_, Cl_2_ |

**Table S3** Chemical reactions, decomposition phases, and decomposition energy, ED, between lithium chlorides (Li_3_MCl_6_), sulfides (Li_3_PS_4_, LGPS, and Li_6_PS_5_Cl), and four cathode materials (LiFePO_4_ (LFP), LiMn_2_O_4_ (LMO), Li(NiMnCo)_1/3_O_2_ (NCM), and LiCoO_2_ (LCO)).^[1]^

| Cathode | SSE | Reaction equation | E_D_ (eV/atom) |
| --- | --- | --- | --- |
| LFP | Li_3_SmCl_6_ | 0.3333 Li_3_SmCl_6_ + 0.6667 LiFePO_4_ → 0.3333 Fe_2_PClO_4_ + 0.3333 SmPO_4_ + 1.667 LiCl | 44 |
|  | Li_3_ErCl_6_ | 0.3333 Li_3_ErCl_6_ + 0.6667 LiFePO_4_ → 0.3333 Fe_2_PClO_4_ + 0.3333 ErPO_4_ + 1.667 LiCl | 38 |
|  | Li_3_InCl_6_ | N/A | 0 |
|  | Li_3_PS_4_ | 0.3 Li_3_PS_4_ + 0.7 LiFePO_4_ → 0.4 Li_4_P_2_O_7_ + 0.2 FePS + 0.5 FeS_2_ | 85 |
|  | LGPS | 0.1463 Li_10_Ge(PS_6_)_2_ + 0.8537 LiFePO_4_ → 0.1951 Li_4_P_2_O_7_ + 0.5122 Li_3_PO_4_ + 0.2439 FePS + 0.1463 GeS_2_ + 0.6098 FeS_2_ | 101 |
|  | Li_6_PS_5_Cl | 0.2857 Li_6_PS_5_Cl + 0.7143 LiFePO_4_ → 0.1818 FePS + 0.02597 P_4_S_7_ + 0.7143 Li_3_PO_4_ + 0.5325 FeS_2_ + 0.2857 LiCl | 136 |
| LMO | Li_3_SmCl_6_ | 0.4 Li_3_SmCl_6_ + 0.6 LiMn_2_O_4_ → 0.06667 Mn_8_Cl_3_O_10_ + 0.6667 MnO_2_ + 1.8 LiCl + 0.4 SmClO | 1 |
|  | Li_3_ErCl_6_ | 0.2857 Li_3_ErCl_6_ + 0.7143 LiMn_2_O_4_ → 0.04762 Mn_8_Cl_3_O_10_ + 0.2857 ErMn_2_O_5_ + 0.4762 MnO_2_ + 1.571 LiCl | 16 |
|  | Li_3_InCl_6_ | N/A | 0 |
|  | Li_3_PS_4_ | 0.5 Li_3_PS_4_ + 0.5 LiMn_2_O_4_ → 0.5 Li_3_PO_4_ + 0.5 Li(MnS_2_)_2_ | 347 |
|  | LGPS | 0.3333 Li10Ge(PS_6_)_2_ + 0.6667 LiMn_2_O_4_ → 0.3333 Li_4_GeS_4_ + 0.6667 Li(MnS_2_)_2_ + 0.6667 Li_3_PO_4_ | 280 |
|  | Li_6_PS_5_Cl | 0.5 Li_6_PS_5_Cl + 0.5 LiMn_2_O_4_ → 0.5 Li_3_PO_4_ + 0.5 Li(MnS_2_)_2_ + 0.5 Li_2_S + 0.5 LiCl | 316 |
| NCM | Li_3_SmCl_6_ | 0.625 Li_3_SmCl_6_ + 0.375 Li_3_MnCoNiO_6_ → 0.02344 Mn_2_CoO_4_ + 0.07031 Li_4_MnCo_5_O_12_ + 0.08594 Li_2_Mn_3_NiO_8_ + 0.2891 NiCl_2_ + 2.547 LiCl + 0.625 SmClO | 51 |
|  | Li_3_ErCl_6_ | 0.5833 Li_3_ErCl_6_ + 0.4167 Li_3_MnCoNiO_6_ → 0.08333 ErMn_2_O_5_ + 0.1667 Er_2_O_3_ + 0.08333 Er_2_Mn_2_O_7_ + 0.08333 Li_4_MnCo_5_O_12_ + 0.4167 NiCl_2_ + 2.667 LiCl | 45 |
|  | Li_3_InCl_6_ | 0.6261 Li_3_MnCoNiO_6_ + 0.3739 Li_3_InCl_6_ → 0.03913 Mn_2_CoO_4_ + 0.1174 Li_4_MnCo5O_12_ + 0.1435 Li_2_Mn_3_NiO_8_ + 0.187 In_2_O_3_ + 2.243 LiCl + 0.4826 NiO | 76 |
|  | Li_3_PS_4_ | 0.6 Li_3_PS_4_ + 0.4 Li_3_MnCoNiO_6_ → 0.6 Li_3_PO_4_ + 0.1333 Li(MnS_2_)_2_ + 0.1333 Co(NiS_2_)_2_ + 0.1333 Co_2_NiS_4_ + 0.1333 MnS_2_ + 0.5333 Li_2_S | 407 |
|  | LGPS | 0.317 Li_10_Ge(PS_6_)_2_ + 0.683 Li_3_MnCoNiO_6_ → 0.317 Li_2_MnGeO_4_ + 0.2277 Co(NiS_2_)_2_ + 0.6339 Li_3_PO_4_ + 0.183 Li(MnS_2_)_2_ + 0.07366 Li_2_SO_4_ + 0.2277 Co_2_NiS_4_ + 1.176 Li_2_S | 344 |
|  | Li_6_PS_5_Cl | 0.4 Li_3_MnCoNiO_6_ + 0.6 Li_6_PS_5_Cl → 0.1333 Co_2_NiS_4_ + 0.1333 Li(MnS_2_)_2_ + 0.1333 Co(NiS_2_)_2_ + 0.1333 MnS_2_ + 1.133 Li_2_S + 0.6 LiCl + 0.6 Li_3_PO_4_ | 364 |
| LCO | Li_3_SmCl_6_ | 0.2857 Li_3_SmCl_6_ + 0.7143 LiCoO_2_ → 0.2857 SmClO + 0.1429 Co_3_O_4_ + 0.1429 Li(CoO_2_)_2_ + 1.429LiCl | 37 |
|  | Li_3_ErCl_6_ | 0.2857 Li_3_ErCl_6_ + 0.7143 LiCoO_2_ → 0.1429 Co_3_O_4_ + 0.2857 ErClO + 0.1429 Li(CoO_2_)_2_ + 1.429 LiCl | 38 |
|  | Li_3_InCl_6_ | 0.2857 Li_3_InCl_6_ + 0.7143 LiCoO_2_ → 0.2857 InClO + 0.1429 Co_3_O_4_ + 0.1429 Li(CoO_2_)_2_ + 1.429 LiCl | 33 |
|  | Li_3_PS_4_ | 0.3143 Li_3_PS_4_ + 0.6857 LiCoO_2_ → 0.3143 Li_3_PO_4_ + 0.02857 Li_2_SO_4_ + 0.2286 Co_3_S_4_ + 0.3143 Li_2_S | 385 |
|  | LGPS | 0.1037 Li_10_Ge(PS_6_)_2_ + 0.8963 LiCoO_2_ → 0.1037 Li_4_GeO_4_ + 0.09959 Co_9_S_8_ + 0.2075 Li_3_PO_4_ + 0.1369 Li_2_SO_4_ + 0.3112 Li_2_S | 325 |
|  | Li_6_PS_5_Cl | 0.3143 Li_6_PS_5_Cl + 0.6857 LiCoO_2_ → 0.3143 Li_3_PO_4_ + 0.6286 Li_2_S + 0.02857 Li_2_SO_4_ + 0.2286 Co_3_S_4_ + 0.3143 LiCl | 348 |

**Diffusion and correlation analysis based on the molecular dynamic simulations of the lithium conductors.**

Ab initio molecular dynamics (AIMD) simulations are conducted using a 2 × 2 × 1 supercell of the studied lithium conductors with a time step of 2 fs. Typical AIMD simulations last over 60-130 ps until the extracted Li-ion diffusion coefficients are converged and show good linearity when fitted to the Arrhenius relation (see below). The first 10 ps are allowed for the system to reach thermal equilibrium before the collection of the structural data at the following time steps. NVT ensemble (with constant volume) is used at different simulated temperatures to speed up the ion hopping process. The diffusivity ($D$) at each temperature is obtained by linear fitting to the calculated mean squared displacement (MSD) from the collected MD data according to

$D=\lim_{t\to\infty} \frac{1}{2dt}\frac{1}{N}\sum_{i=1}^{N} \left\langle\left[ r_{i}\left( t \right)-r_{i}\left( 0 \right) \right]^{2} \right\rangle$,

where $\left[ r_{i}\left( t \right)-r_{i}\left( 0 \right) \right]$ is the displacement vector of Li^+^ at time t.^[2]^ The ionic conductivity is then calculated from the Nernst-Einstein relation

$$\sigma=D\left( \frac{Ne^{2}}{k_{B}T} \right)$$

with $N$ the number of ions per cm^3^. Other symbols have their customary meanings. The Arrhenius relationship

$$D=Aexp\left( \frac{E_{a}}{k_{B}T} \right)$$

is used to fit the diffusivities at different temperatures and extrapolate to the value at room temperature. The pre-factor $A$ and activation energy $E_{a}$ are the fitting parameters in the relation. The diffusivity that measures the diffusion of the charge center of all the Li-ions (rather than individual Li-ion) in the system is defined as

$D_{c}=\lim_{t\to\infty}\frac{1}{2dt}\left\langle\left[ \frac{1}{N}\sum_{i=1}^{N} r_{i}\left( t \right)-\frac{1}{N}\sum_{i=1}^{N} r_{i}\left( 0 \right) \right]^{2} \right\rangle$.

**Electrochemical and interface stability.**

The phase equilibria are evaluated along the energy minimum using the decomposition energy $\Delta E_{D}$. It is determined by comparing the energy of all relevant phases in their compositional space. The grand potential phase diagram identifies the phase equilibria $C_{eq}$ ($C,\mu_{Li}$) of a given phase with the composition $C$ in equilibrium with the chemical potential $\mu_{Li}$ of element Li.^[3, 4]^ The decomposition reaction energy at a given chemical potential $\mu_{Li}$ of element Li is calculated as:

$\Delta E_{D}^{open}\left( phase, \mu_{Li} \right)=E_{eq}\left( C_{eq}\left( C,\mu_{Li} \right) \right)-E\left( phase \right)-\Delta n_{Li}\cdot\mu_{Li}$,

here the chemical potential *µ*_Li_ is referenced to Li metal.

The interface pseudo-binary reaction energy is calculated as:

$$\Delta E_{D}\left( SSE, electrode,x \right)=$$

$E_{eq}\left( C_{interface}\left( C_{SSE},C_{electrode},x \right) \right)-E_{interface}(SSE,electrode,x)$,

where $C_{SSE}$ and $C_{electrode}$ are the compositions of SSE and electrode materials, normalized to one atom per formula. The $x$ is the molar fraction of the SSE.^[5, 6]^ The energies of materials are obtained from MP database.^[7]^

**Reference**

[1] K. Kim, D. Park, H.-G. Jung, K. Y. Chung, J. H. Shim, B. C. Wood, S. Yu. *Chem. Mater.* **2021,** *33*, 3669.

[2] X. He, Y. Zhu, A. Epstein, Y. Mo. *NPJ Comput.* **2018,** *4*, 18.

[3] S. P. Ong, L. Wang, B. Kang, G. Ceder. *Chem. Mater.* **2008,** *20*, 1798.

[4] Y. Zhu, X. He, Y. Mo. *ACS Appl. Mater. Interfaces* **2015,** *7*, 23685.

[5] Y. Zhu, X. He, Y. Mo. *J. Mater. Chem. A* **2016,** *4*, 3253.

[6] Y. Zhu, X. He, Y. Mo. *Advanced Science* **2017,** *4*, 1600517.

[7] A. Jain, S. P. Ong, G. Hautier, W. Chen, W. D. Richards, S. Dacek, S. Cholia, D. Gunter, D. Skinner, G. Ceder, K. A. Persson. *APL Mater.* **2013,** *1*, 011002.
